# Using daily text messages to improve adherence to infant micronutrient powder (MNP) packets in rural western China: A cluster-randomized controlled trial

**DOI:** 10.1371/journal.pone.0191549

**Published:** 2018-01-19

**Authors:** Xu Wang, Renfu Luo, Chengfang Liu, Linxiu Zhang, Ai Yue, Alexis Medina, Scott Rozelle

**Affiliations:** 1 Education Global Practices (East and South Africa), the World Bank Group, NW, Washington DC, United States of America; 2 China Center for Agricultural Policy, School of Advanced Agricultural Sciences, Peking University, Haidian District, Beijing, China; 3 Center for Chinese Agricultural Policy, Institute of Geographical Sciences and Natural Resources Research, Chinese Academy of Sciences, Chaoyang District, Beijing, China; 4 Center for Experimental Economics in Education (CEEE), Shaanxi Normal University, Xi’an, Shaanxi, China; 5 Freeman Spogli Institute for International Studies, Stanford University, Stanford, United States of America; TNO, NETHERLANDS

## Abstract

**Objective:**

To evaluate the effectiveness of daily text messages as a means to improve caregivers’ adherence to infant micronutrient powder (MNP) in rural Shaanxi Province of China.

**Methodology:**

638 infants aged 6–11 months in 234 villages were involved in a cluster-randomized controlled trial (RCT). All caregivers were given free infant MNP packets at baseline in April 2013 and the follow-up survey was in July 2013. We randomly assigned 318 infants in 117 villages to treatment group (receiving daily text message) and 320 infants in the other 117 villages as control group.

**Results:**

On average, daily text messages increased the number of MNP packets fed (marginal effect = 4.63; 95% confidence interval (CI) = 0.16, 9.10). The text message is more likely to increase the consumption of MNP packets if the primary caregiver was the mother (marginal effect = 12.19; 95% CI = 0.69, 23.68). Receiving the text message appears to significantly increase the likelihood of full adherence when the primary caregiver can either check (odds ratio = 2.93; 95% CI = 1.34, 6.40) or knows how to send (odds ratio = 3.26; 95% CI = 1.53, 6.97) text messages.

**Conclusion:**

Daily text messages improved the consumption of infant MNP packets. However, the impact was not large enough to increase the probability of caregivers being fully adherent to the feeding instruction, which is to feed 5–7 packets per week as recommended. In addition, when the mother is the caregiver and when the caregiver can check or knows how to send text messages there is greater adherence by the primary caregivers.

**Trial registration:**

http://www.isrctn.com/ISRCTN44149146

## Introduction

Malnutrition kills or disables millions of children every year, and prevents millions more from reaching their full intellectual and productive potential [1–2]. Early childhood malnutrition is associated with adverse outcomes in school-aged children and adolescents, including an increased prevalence of conduct problems (such as bullying and cheating) and aggressive behaviors [3]. Poor nutrition in utero and during early childhood has also been shown to have negative consequences for adult health and human capital [2]. Children, undernourished between conception and age two, are at higher risk of impaired cognitive development, which adversely affects a country’s productivity [4].

In rural China, there are still a significant number of infants lacking access to a nutritious diet. The Chinese Food and Nutrition Surveillance System found that anemia prevalence among infants aged 6–11 months in rural areas was around 28% in 2010 [5]. Other more geographically focused studies have found anemia prevalence ranging from 22.6% in Guangxi Province [6] to 58.2% in Gansu Province [7] among the same age group.

Micronutrient powder (MNP) provision is considered as an effective way of reducing malnutrition. Provision of MNP to infants and children under two years of age reduced anemia by 31% when compared with placebo group [8]. MNP is regarded as an important strategy for preventing anemia in Asia, Africa, and in prioritized communities in developed countries [9]. In the past, MNP provision was also shown to improve infant nutritional status in China [10–12].

While MNP has the potential for reducing infant malnutrition, there are indications that non-adherence is a barrier to treatment success. It has been estimated that 20% to 50% of patients do not adhere to their prescribed therapies [13]. Dissemination efforts of medical treatment are often weak, and adoption rates remain low [14]. For instance, two studies conducted in Peru on MNP found that many patients discontinued the MNP supplementation during the intervention and did not adhere to the proper proscribed trial protocol [15–16].

Non-adherence to medical treatment has negative outcomes including increased morbidity and consumption of health care resources, especially in some resource-limited settings [17]. It is in essence a behavioral problem that may be attributed to many factors. Forgetfulness is a barrier that has been found to be associated with non-adherence in similar intervention studies [18–19]. For example, a study on the adherence to the ferrous sulfate treatment in children younger than 42 months found that in nearly 40% of the time there was non-adherence that was due to forgetfulness [20].

Luckily advances in communication technology open possibilities in innovation to help address the problem. The number of mobile (cell) phone users is rapidly expanding (4.5 billion mobile phone subscribers are expected worldwide by 2020) [17]. Particularly in China, a large proportion of households (around 90%) have mobile phones, even in rural areas [21]. Mobile technology has the potential to be used in health systems worldwide.

Compared to other modes of communication, text messages have many advantages such as timeliness, low cost, and high reliability [22]. It has been widely regarded as a feasible and effective approach to boost the adherence rate of medical treatment hospital patients [17, 19, 23–24]. It was also thought to increase the attendance rate of follow-up care among children with or exposed to HIV [25]. Researchers showed that text messages have the potential to improve smoking cessation rates [26]. Specifically in China, health information transmitted via text messages was shown to positively affect rural students’ health and education outcomes [25, 27].

In spite of these encouraging results, there are a limited number of high-quality text messaging intervention studies. Besides, many existing studies lack adequate sample size to provide sufficient statistical power [28]. Using a cluster-randomized controlled trial (RCT) design, we aimed to explore if daily text messages would enhance the adherence of rural caregivers to infant MNP feeding. We had three objectives. First, we studied the marginal effect of text messages on the quantity of MNP packets caregivers fed. Second, we analyzed the impact of the text message on the odds ratio of caregivers being fully adherent, or feeding 5–7 packets per week as recommended. Third, we examined whether the impact of the text message varied among different caregivers.

## Methods

### Sampling

We conducted a cluster-RCT using villages as clusters in southern Shaanxi Province in the northwest part of China. One of the strengths of the clustered-RCT design is that it helps to reduce spill-over effect. A total of 638 infants aged 6–11 months of 234 villages at 11 nationally designated poor counties were involved. On average, there were 21 villages in each county and around three infants at each village. This trial is registered with ISRCTN (ISRCTN44149146). The protocol was approved by the institutional review board of Stanford University (No. 25734) and the ethical review board of Sichuan University (No. 2013005–01). We obtained written consent from participants at the beginning of the intervention according to the protocol. The participant had the opportunity to opt out of the study at any time.

We determined the sample size by power calculations before enrollment using Optimal Design, a software developed by University of Michigan [29]. The power to detect a difference in adherence between the treatment group and the control group in a cluster-RCT depends on the following four parameters in our case: a.) the number of infants per village, b.) the number of villages, c.) the intra-cluster correlation of adherence, and d.) the minimum detectable effect.

Based on a previous study [30], we assumed a minimum detectable effect of 0.3 SD, three infants per village, and an attrition rate of 10%. The calculation based on these parameters indicated that we need 105 villages per group to detect a standardized effect of 0.3 SD at 80% power given a significance level of 0.05 and an intra-cluster correlation of 0.2. We added 12 villages to each group to overpower the study when the budget allowed.

In March 2013, we obtained a complete official list of all villages in each township. The townships that housed the county seat were excluded. Then, we randomly selected 234 villages from the listed township and enrolled all families with infants who were 6–11 months old. We require the infant’s household to have at least one cell phone to be included. Half of the sample (117 villages and 318 infants) were randomly selected as the text message group after baseline survey in April 2013, who would receive free MNP packets as well as daily text messages. The other half (117 villages and 320 infants) were assigned as the control group, who would only receive free MNP packets. After assignment, caregivers did not know whether or not they were in an experiment.

### Intervention

We used a Heinz-produced micronutrient powder called NurtureMate. The powder was tasteless and contained a mix of iron; zinc; vitamins A, C, D, B1, B2, B6, and B12; and folic acid. Certified by State Drug and Food Administration, NurtureMate is recommended for children aged 6 to 36 months. It is supposed to be mixed into infant’s food. Caregivers were instructed to feed their child one packet per day, and at least five days a week. S1 Table shows the MNP packet’s micronutrient content as supplemental material along with the percentage of recommended daily amounts for children 6–12 and 13–36 months of age.

The enumerators, who were trained by nutrition experts distributed the MNP packets and trained the caregivers. The infant caregivers were given a three-month supply of MNP packets, or 90 packets in April 2013. The total trial period was 69 days on average; therefore, the households received a sufficient supply. The one-on-one enumerator training session focused on four areas: (1) The harm caused by malnutrition in poor rural areas; (2) feasible methods for reducing malnutrition through a balanced diet, diet supplements, etc.; (3) the effect of MNP packets on reducing malnutrition; and (4) how to use MNP packets. The caregivers also were given free booklets that covered the training material. To ensure the accuracy of the self-reported consumption of MNP packets, we instructed caregivers to put used empty packets in a pre-prepared plastic storage envelope and the not yet used ones in another envelope. Enumerators tallied the unused and the empty packets to see if the sum equals 90.

We partnered with a cellular communications service provider based in Shaanxi Province (China Mobile Communications Corporation), from which caregivers in the text message group would receive daily message reminders, with modifications in the text content every day. The idea of the text message was always to remind the caregivers to feed the infant with the MNP packet every day, with slight modifications in wording of the text in seven days of the week. The following measures were taken to make sure the text message get properly conveyed: first, we made sure that the text is concise yet carry the core message of remembering to feed the infant with the MNP packet; second, the text did not exceed 45 Chinese characters so that caregivers could read the entire message without scrolling; third, in consideration of caregivers who were illiterate or with little education, we told the primary caregiver the message content and sent testing messages during the training period; fourth, in the case of illiterate caregivers, we also told the secondary caregiver the message content and encouraged the infant’s primary caregiver to communicate with the secondary caregiver on received messages; fifth, the message reminding the caregiver to feed the infant was sent by a fixed telephone number so that the recipient could recognize the reminder text message without checking the message content. The message was usually sent at 9 AM every day during the treatment period, and recipients were not required to reply. The content of the message in Chinese and its English translation is shown in S2 Table.

The trial region was mostly mountainous, and villages were far away from each other. The interactions between families in different villages are infrequent. Within a village, we enrolled all households with infant aged 6 to 11 months. A majority of our infants have no siblings of a similar age. There was a low chance that the caregivers fed the MNP packets to other children; therefore, a spillover effect between the treatment and control group was unlikely.

### Data

The baseline survey was conducted in April 2013 (S1 File). Enumerators visited households to survey the infant’s primary caregiver. Detailed information about the infant’s parents and all other family members who lived at home for more than three months in the recent year were collected, including information about the infant, the parents, the primary caregiver, and the household. We also collected the cell phone numbers and family members’ text message habits.

The follow-up survey was conducted in July 2013 (S2 File). The self-reported number of MNP packets fed was cross-checked with the number of empty packets and the unused MNP packets put in the envelopes.

### Statistical methods

We conducted the statistical analysis using Stata version 13.0 (StataCorp, College Station, TX). P-Value less than 0.05 was considered statistically significant. Our primary outcome variable was the number of MNP packets fed over the trial period. The secondary outcome variable was a dummy variable of being “Adherent,” which was defined as feeding the infant 5–7 packets per week according to the self-reported data. The dummy variable was determined using the average weekly consumption of infant MNP packets over the duration of the trial period.

A linear multivariate regression model and a logistical multivariate regression model were respectively built to examine the average treatment effect in an intention-to-treat analysis. Both models controlled for observable baseline characteristics of the infant, the primary caregiver and the household, as well as the primary caregiver’s text message practice. The county fixed effects were also controlled for. In all analysis, standard errors were clustered at the village level. Besides average treatment effect, interaction terms between the treatment variable and each control variable were respectively generated and added to each model to explore whether the text message’s impact among different groups of caregivers varied.

## Results

**Fig 1:**
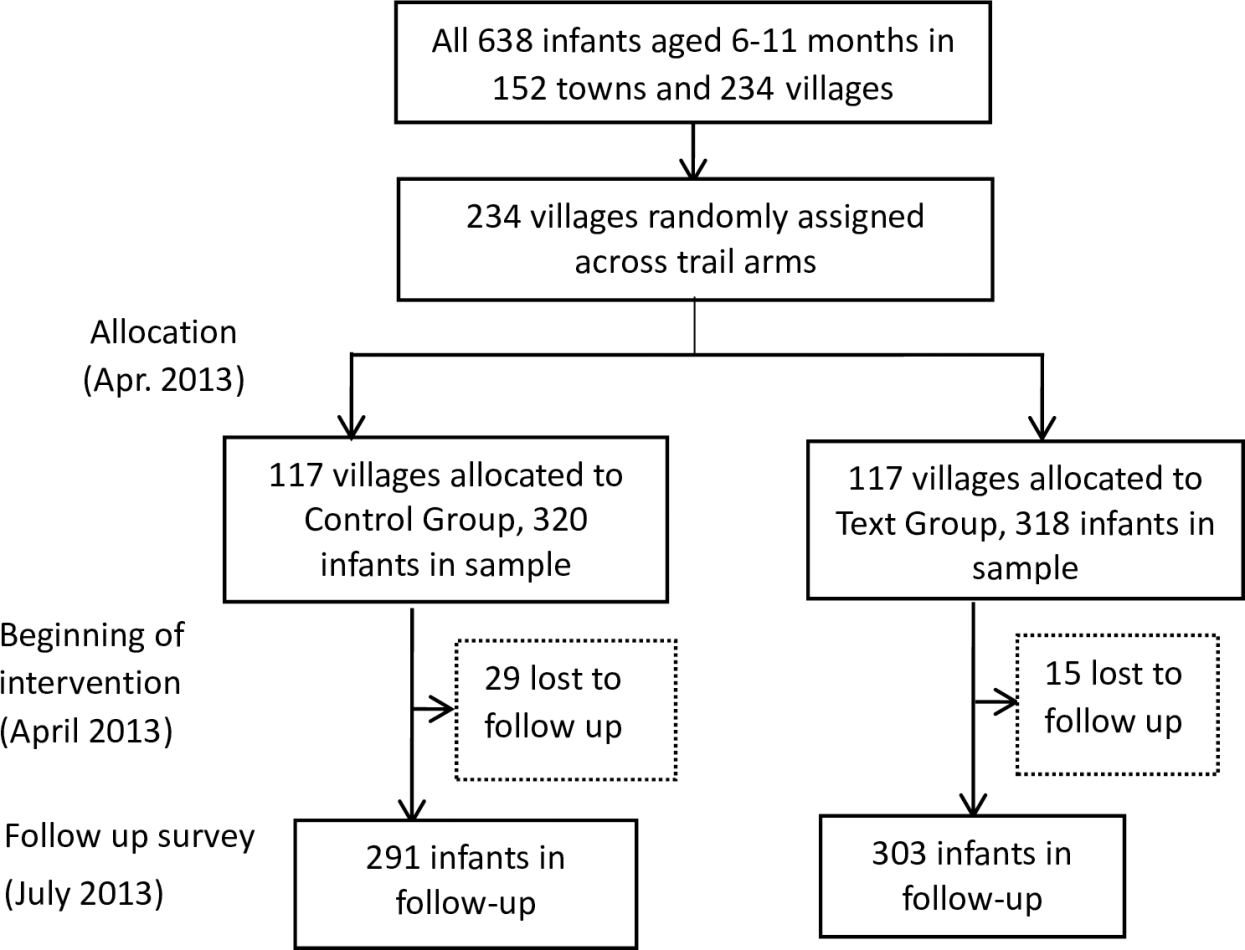
Trial of text message campaign on the adherence of rural caregivers in feeding infants with free MNP packets, comprising the control group and the text group (April 2013-July 2013): Rural Shaanxi Province, China.

Fig 1 shows our study design. During the whole intervention between April 2013 and July 2013, 15 infants (4.72%) of the text group and 29 infants (9.04%) of the control group were lost to the follow-up survey. In total 594 infants were analyzed (303 infants in the text group and 291 infants in the control group).

All infants of both trial arms had caregivers with mobile phones to receive text messages. Table 1 shows the descriptive statistics for major variables at baseline for all participants (n = 638).

**Table 1:** Baseline characteristics of sample infants, caregivers, household social economic status, and primary caregivers’ text message practice by trial groups: Rural Shaanxi Province, China, 2013.

Characteristics	Control Group^1^ (n = 320)	Text message Group^2^ (n = 318)
***Infant characteristics***		
Age in months	9.24±1.78	9.05±1.71
Boys (%)	46.6 (149)	47.8 (152)
***Caregiver and mother characteristics***		
Mother is primary caregiver (%)	78.4 (251)	82.7 (263)
Maternal education ≤ 6 years (%)	31.9 (102)	24.5 (78)
***Socio economic status***		
Families received social security support (%)	25.0 (80)	21.4 (68)
***Text message practice***		
Would check text message (%)	74.7 (239)	78.9 (251)
Knows how to send text message (%)	68.1 (218)	75.8 (241)

*Note*. Total Sample was n = 638

Data are presented as mean ± SD for continuous variable or % (*n*) for categorical variables.

^1^Provide free MNP packets.

^2^ Provide free MNP packets and text message reminder every day.

On overage the text message group consumed 41.57 packets while the control group consumed 36.18 packets. The rate of full adherents in the text message group was 46.86% compared to 40.94% in the control group.

**Fig 2:**
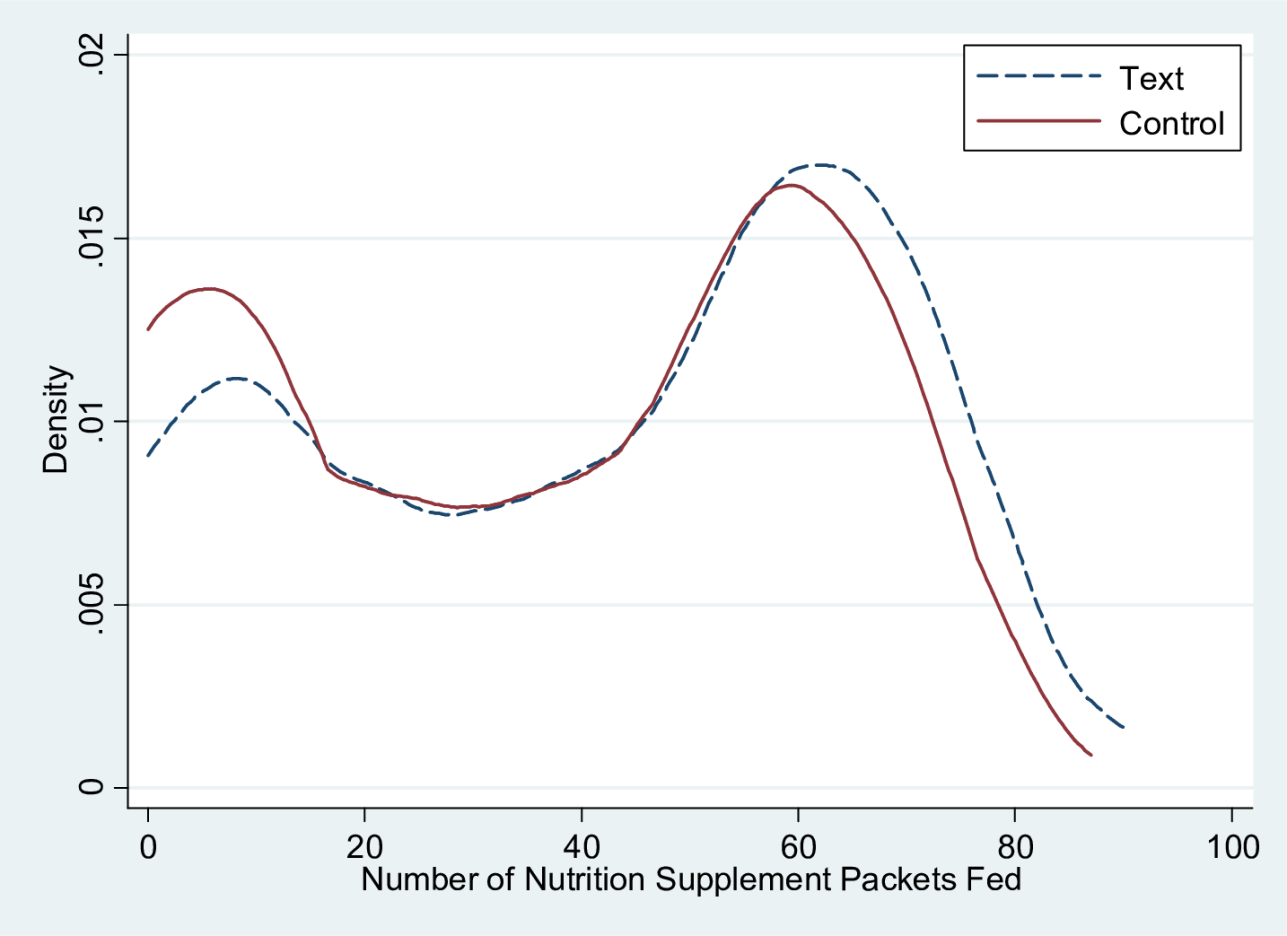
Density distribution curve of the number of MNP packets fed by trial arms: Rural Shaanxi Province, China.

Fig 2 shows the density distribution curve of the number of MNP packets consumed throughout the experiment by trial arms. As the figure shows, the text message group has greater probability density in the high consumption spectra compared with the control group. On average, the text message reminders encouraged caregivers to feed more MNP packets.

Table 2 shows the impact of the text message on caregiver adherence. Here the marginal effect is defined as the amount of change in the outcome variable brought by one unit change in one independent variable of interest while holding other covariates constant [31]. After adjusting for covariates, we found that on average the text message increased infants’ consumption of MNP packets by 4.63 packets (marginal effect = 4.63; significant at 0.05 and 95% confidence interval [CI] = 0.16, 9.10). However, on average the text message failed to significantly increase the probability of caregivers being fully adherent to feeding instructions. As Table 2 indicates, though the odds ratio of the treatment variable is greater than 1, it is not significant at 0.05 (odds ratio = 1.25; 95% CI = 0.88, 1.79).

**Table 2:** Intent-to-treat analysis of the impact of daily text messages on caregiver feeding adherence: Rural Shaanxi Province, 2013.

Number of micronutrient supplement packets fed^1^	Marginal Effect (95% CI)	*P*
Text message group	4.63 (0.16, 9.10)	0.042
Full Adherence^1^^,^^2^	Odds Ratio (95% CI)	*P*
Text message group	1.25(0.88, 1.79)	0.218

*Note*. CI = confidence interval. Total Sample was n = 638.

^1^Adjusted for infant’s age, gender, mother as the infant’s primary caregiver, mother finished primary school education, households received social security support, primary caregiver would check text messages, primary caregiver knows how to send text messages, as well as county fixed effects. Clustering is at the village level. Control group = 0, and Text Group = 1 in this model.

^2^Caregiver adherence was measured by administration to child 5 to 7 MNP packets per week (yes = 1, no = 0).

We also explored the heterogeneous impact of the text message by characteristics of infants (age groups; the gender), caregivers (whether mother was the primary caregiver; whether mother had primary school education), and text message practice (whether the primary caregiver would check text messages; whether the primary caregiver knows how to send text messages). Panel A of Table 3 reports the coefficient of each interaction term significant at 0.05. It suggests that the text message would be more likely to increase the consumption of MNP packets if the primary caregiver was the mother (marginal effect = 12.19; 95% CI = 0.69, 23.68). Besides, if the primary caregiver would check text messages, she/he was also more likely to respond to the message (marginal effect = 9.72; 95% CI = 0.20, 219.25). The same holds for primary caregivers who knows how to send text messages (marginal effect = 14.68; 95% CI = 5.37, 23.98). In fact, as suggested by Panel B of Table 3, receiving the text message significantly increases the likelihood of full adherence when the primary caregiver can either check (odds ratio = 2.93; 95% CI = 1.34, 6.40) or know how to send (odds ratio = 3.26; 95% CI = 1.53, 6.97) text messages.

**Table 3:** Heterogeneous impact of daily text messages: Rural Shaanxi Province, 2013.

*Panel A*		
Number of micronutrient supplement packets fed	Marginal Effect (95% CI)	*P*
Text message group and Mother was primary caregiver^1^	12.19 (0.69, 23.68)	0.038
Text message group and Primary caregiver would check messages^1^	9.72(0.20, 19.25)	0.046
Text message group and Primary caregiver knows how to send messages^1^	14.68(5.37, 23.98)	0.002
*Panel B*		
Full adherence	Odds Ratio (95% CI)	*P*
Text message group and Primary caregiver would check messages^1^^,^^2^	2.93(1.34, 6.40)	0.007
Text message group and Primary caregiver knows how to send messages^1^^,^^2^	3.26(1.53, 6.97)	0.002

*Note*. CI = confidence interval. Total Sample was n = 638.

^1^Adjusted for treatment assignment, infant’s age, gender, mother as the infant’s primary caregiver, mother finished primary school education, households received social security support, primary caregiver would check text messages, primary caregiver knows how to send text messages, as well as county fixed effects. Clustering is at the village level. Control group = 0, and Text Group = 1 in this model.

^2^Caregiver adherence was measured by administration to child 5 to 7 MNP packets per week (yes = 1, no = 0).

## Discussion

All MNP programs must develop and deliver behavior change communication strategies in order to generate demand, ensure continued high coverage, and reinforce appropriate use of MNP in the target population [32]. Studies show that the frequency and quality of communication between the caregiver and the MNP channel distributor is positively associated with overcoming MNP feeding barriers such as perceived changes in texture/taste/smell of foods in which MNP were added, lack of availability of semisolid food, side effects (change in color and consistency of stool; vomiting), and superstitions and disbeliefs about the value and benefits of the product [33–37]. Examples of behavior change communications in order to boost adherence are many, such as counselling caregivers on MNP side effects [38], providing mothers with a reminder card to support them in establish a routine for children MNP intake [39], paying home visits to encourage the use of the MNP, using mass media to increase MNP dissemination effort [40]. Compared with all these methods text messaging appears to be an innovative one and has great potential. It has been used in improving adherence for treatment including MNP feeding and has been widely regarded as a feasible and effective approach [17, 19, 23–24, 41].

Despite the availability of studies on message reminders’ effect on caregiver feeding practices, research has been largely on the effect of message reminders on school aged children. There is little evidence on the impact of text messages on infant nutrition. Besides, similar message reminder studies on MNP have seldom explored the underlying reasons or the heterogeneous effects of the intervention.

Our findings show that on average the text message to caregivers increased the consumption of MNP packets. However, the text messages did not increase the probability of full adherence, or feeding 5–7 packets per week on average as recommended. The text messages might have increased infants’ consumption of MNP packets by addressing the forgetfulness of caregivers. Addressing forgetfulness had previous been tested in other similar studies [18–19]. The text message could work as a stimuli to feed the infant and reinforce the feeding behavior of caregivers. In this way, a habit would be acquired [23]. The text messages could also provide an important reminder, especially when caregivers encounter disruption to their routine adherence [41].

On the other hand, text message’s impact was not large enough to convert non-adherence to full adherence, indicating that there are other factors that the messages did not address. One such factors might be the attitude caregivers towards the MNP packets. If there were issues involving the willingness to adhere, such as cognition problems, attitudes or belief, then the adherence rate would be low [42–43]. Studies have shown that some of the caregivers in rural China lacked proper information and were not willing to feed their child unfamiliar foods [44]. If caregivers were suspicious about MNP packets and lacked the incentive to adhere, the text message would fail to work as a reminder.

We found the text messages was working better for certain caregiver groups.

First, caregivers who would check or knows how to send text messages significantly increased the consumption of MNP packets by their infants and were likely to exhibit full adherence. Research on mobile marketing indicates that the interactions between customers and the company via mobile technology increase consumers’ trust and perceived relevance of the advertised information [28, 45]. The novelty of receiving messages can lead to an increased likelihood of purchase [46]. Such a positive effect could only be realized if caregivers had a working proficiency with text message functions. We thought about the possibility of caregivers cannot read text messages due to illiteracy, but our data showed that illiteracy is not a big problem in our study. The literacy rate of primary caregivers is around 92%. Only 5% of the sample households have both illiterate primary and secondary caregivers, and there was not significance difference between the treatment and comparison groups. Besides, there were no significant difference between literate and illiterate primary caregivers on the consumption of MNP packets. This finding is particularly important given the rapid development of mobile technology because text message campaigns can be more frequently utilized in health interventions.

Second, we found that when primary caregiver was the mother, the response was higher. The infants were mostly taken care of by grandparents if the primary caregiver was not mother. This indicates that it might be harder for text messages to change the infant feeding behaviors of elderly caregivers. Grandparent childcare is a traditional and common practice in China, where intergenerational ties are strong and collective interests are emphasized over individual interests. This reality is even more pronounced in rural China, where the grandparents and grandchildren are left at home by their children, who migrate to the cities for work a migratory middle generation [47–48]. While we do not have data to prove this, during the field work we observed that compared to their parents, the infants’ grandparents were usually less educated, relied more on their past feeding experiences, and were more conservative about new feeding practices. Grandparents, therefore, might be more suspicious about MNP packets and less responsive to the text messages. Additionally, the grandparents were often less proficient in using mobile phones and were less likely to check and read messages as regularly as parents, which might also have contributed to the low responsiveness.

Although our work contributes to the understanding of the impact of text messages on infants’ consumption of MNP packets, several limitations should be recognized. First, we used self-reported number of MNP packets fed as the main indicator of adherence, and there is evidence that such measurement might overestimate adherence rate [49]. By instructing caregivers to put empty packets in the envelope we attempted to address the measurement error, but it would be preferable if we could track people’s behaviors by real-time electronic monitoring devices [18]. Second, our messages were designed to serve as reminders for caregivers, and little attention was paid to messages’ pattern and content. As shown in S2 Table, although caregivers received modified messages, the message content did not vary much throughout the trial period. Third, the text message was often sent at a fixed time of the day (9 AM). As the frequency, content, and the timing of messages influence the recipients’ perceptions of the intervention [50], it was possible the recipient got bored with one message a day, which could contribute to the partial adherence. In future studies researchers should consider personalizing the message content and varying the time of sending the message. Finally, though we set up hotlines and asked caregivers for feedback on message reminders and MNP packets, we nonetheless likely do not fully understand the perception or attitudes of our subjects towards the intervention. Qualitative studies including in-depth interviews and focus groups could further explore these issues.

## Conclusion

On average, the text message reminders increased the consumption of MNP packets by the infants. However, the impact was not large enough to increase the probability of caregivers of being fully adherent to feeding instruction, which is to feed 5–7 packets per week as recommended. We also found that caregivers who were familiar with text message functions and mother caregivers are more responsive to text message reminders.

## Supporting information

S1 Table Composition of the NurtureMate home fortification powders. (DOCX)

S2 Table List of daily text messages sent as part of the project, in original Chinese and English. (DOCX)

S1 File Baseline household survey form. (DOCX)

S2 File Endline household survey form. (DOCX)

S3 File Research protocol. (PDF)

S4 File CONSORT Extension for cluster trials checklist. (DOCX)

S5 File Data for the study. (DTA)

S6 File Do file for the study. (DO)
