# Supplementary material for: Using daily text messages to improve adherence to infant micronutrient powder (MNP) packets in rural western China: A cluster-randomized controlled trial
Source: PLoS One. 2018 Jan 19;13(1):e0191549. doi: 10.1371/journal.pone.0191549 (PMC5774801; doi:10.1371/journal.pone.0191549)
Supplement: S1 Table — (DOCX) [file pone.0191549.s001.docx]

**S1 Table. Composition of the NurtureMate home fortification powders^a^**

| Nutrients | Unit | Average content  per gram | RDA^b^,% | | |
| --- | --- | --- | --- | --- | --- |
|  |  |  | 6-12 month | 13-36 month | |
| Iron | Mg | 6.0 | 60% | | 50% |
| Zinc | Mg | 4.80 | 60% | | 53% |
| Vitamins A | μgRE | 200 | 50% | | 40% |
| Vitamins C | Mg | 50.0 | 100% | | 83% |
| Vitamins D | Μg | 5.0 | 50% | | 50% |
| Vitamins E | Mg | 1.55 | 52% | | 39% |
| Vitamins B_1_ | Mg | 0.30 | 100% | | 50% |
| Vitamins B_2_ | Mg | 0.50 | 100% | | 83% |
| Vitamins B_6_ | Mg | 0.30 | 100% | | 60% |
| Vitamins B_12_ | Μg | 0.5 | 100% | | 56% |
| Folic acid | Μg | 66 | 140% | | 75% |
| Energy | KJ | 15 | － | | － |
| Protein | G | 0 | － | | － |
| Fat | G | 0 | － | | － |
| Carbohydrate | G | 0.9 | － | | － |

^a^NurtureMate can be mixed in with either water or complementary foods, such as porridge, and is recommended for babies aged 6-36 months and taking 5 packets per week or one day one packet.

^b^RDA: recommended daily allowance for Chinese infants.
