# Supplementary material for: Using daily text messages to improve adherence to infant micronutrient powder (MNP) packets in rural western China: A cluster-randomized controlled trial
Source: PLoS One. 2018 Jan 19;13(1):e0191549. doi: 10.1371/journal.pone.0191549 (PMC5774801; doi:10.1371/journal.pone.0191549)
Supplement: S2 Table — (DOCX) [file pone.0191549.s002.docx]

**S2 Table. List of daily text messages sent as part of the project, in original Chinese and English**

| 1、记得给宝宝吃一小袋营养包！营养包可以可添加到已做好的粥、菜、面条、水、奶、果汁或汤中。  Remember to give the baby a nutritional supplement packet! It can be added to porridge, vegetables, noodles, water, milk, juice, or soup. |
| --- |
| 2、别忘了今天要在给宝宝吃的东西里加一小袋营养包，它能让您的宝宝长得更高、更健壮！  Don’t forget to add a nutritional supplement packet to baby’s food today! It can make your baby grow taller and stronger! |
| 3、记得今天给宝宝吃一小袋营养包！添加营养包时，注意粥、菜、面条、水、奶、果汁或汤不能太烫了！  Remember to give your baby a nutritional supplement packet today! When you add the supplement, make sure that the porridge, vegetables, noodles, water, milk, juice, or soup isn’t too hot! |
| 4、提醒您今天在给宝宝吃的东西里加一小袋营养包，它能让您的宝宝变得健康，少生病！ Please remember to add a nutritional supplement packet to baby’s food today. It can make the baby healthier and less likely to get sick! |
| 5、宝宝吃营养包的时间又到了！别忘了今天在宝宝吃的东西里加一小袋营养包。 It’s time for Baby’s vitamin packet again! Don’t forget to add a vitamin packet to Baby’s food today. |
| 6、记得今天要在给宝宝吃的东西里加一小袋营养包，它能让您的宝宝变得更活泼、更聪明！ Remember to add a nutritional supplement packet to baby’s food today. It can make your baby smarter and more active! |
| 7、营养包，营养全，宝宝吃了更健康！ 提醒您在宝宝吃的汤或者粥里拌上一小袋营养包吧。 Nutritional supplement packet helps to keep a nutritious diet for your baby. Baby will be healthier after taking it! Please remember to mix a nutritional supplement packet into baby’s soup or porridge. |
