# Supplementary material for: Using daily text messages to improve adherence to infant micronutrient powder (MNP) packets in rural western China: A cluster-randomized controlled trial
Source: PLoS One. 2018 Jan 19;13(1):e0191549. doi: 10.1371/journal.pone.0191549 (PMC5774801; doi:10.1371/journal.pone.0191549)
Supplement: S1 File — (DOCX) [file pone.0191549.s003.docx]

**Baby Nutrition Program Questionnaire for Parents（need responses from persons who are first and second in charge of infant’s diet and nutrition）**

**_____Province____City（District）______County_______Town_______Village_____Group（natural village），Name of the infant ；**

**Name of the infant’s father： ；Name of the infant’s mother： ；respondent’s name: ___________，personal code: _____**

**Surveyor’s name:_____________ questionnaire code：□□□□□□□，**

Here survey the infant’s parents, and all family members who lived at home for more than three months in the recent one year

**Name of the person first-in-charge of the infant’s diet and nutrition __________，personal code_______；**

**Name of the person second-in-charge of the infant’s diet and nutrition __________，personal code_______**

| Personal code |  |  |  |  |  |  |  |  |  |  |  |
| --- | --- | --- | --- | --- | --- | --- | --- | --- | --- | --- | --- |
|  | gender：  1=male，2=female | Relations with the infant：  1=father，2=mother，3=grandpa on father’s side，  4=grandma on father’s side，5=grandpa on mother’s side，6=grandma on mother’s side，7=older brother（sister）， 8=younger brother(sister)， 9=other cousins，10=uncle(aunt）， 11=others(**please illustrate**) | Nationality：  1=Han，  2=Hui，  3=Mongol，  4=others(please illustrate) | age（solar calendar） | Education level：  0=never went to school，  1=primary school，  2=junior high school，  3=high school/technical secondary school，  4=junior college，  5=college and above | Marital status：  1=married，  2=divorced，  3=widowed，  4=single | Health status：  1=very healthy，2=heathy，  3=normal, not good or bad，4=not in good health condition，5=in a really bad health condition，6=other，please illustrate | Main profession（occupation）：  1=student，2=farmer，  3=migrant worker，4=small business owner，5=civil servant，6=babysitting at home，7=others，please illustrate | Working location：  1=within the town，  2=another town in the county，3=another county in the province，4=outside the province | Still living at home?  1=yes，  2=no | Time living at home in 2012（months） |
| 101 |  |  |  |  |  |  |  |  |  |  |  |
| 102 |  |  |  |  |  |  |  |  |  |  |  |
| 103 |  |  |  |  |  |  |  |  |  |  |  |
| 104 |  |  |  |  |  |  |  |  |  |  |  |
| 105 |  |  |  |  |  |  |  |  |  |  |  |
| 106 |  |  |  |  |  |  |  |  |  |  |  |
| 107 |  |  |  |  |  |  |  |  |  |  |  |
| 108 |  |  |  |  |  |  |  |  |  |  |  |

| Personal code |  |  |  |  |  |  |  |  |  |
| --- | --- | --- | --- | --- | --- | --- | --- | --- | --- |
|  | Is he/she has cellphone?  （1=yes，2=no（jump to the next person），3=uncertain） | What is his/her phone number?(please fill”999”if you don’t know) | Does she/he often turn off the phone?  1=yes，2=no | How many text messages per week would he/she receive from strangers (such as news, advertisement）?  (1=0，2=1-5，3=6-10，4=11 and above) | Would he or she know how to read the text messages?（1=yes，2=no，3=uncertain） | Would he read messages from strangers? (Such as news, advertisements)? (1=yes，2=no，3=I don’t know) | Des she/he know how to send text messages?  (1=yes，2=no，3=uncertain) | Has he/she changed his/her phone number in the recent half year?  1=yes，2=no | Is he/she literate?（1=yes，2=no） |
| 101 |  |  |  |  |  |  |  |  |  |
| 102 |  |  |  |  |  |  |  |  |  |
| 103 |  |  |  |  |  |  |  |  |  |
| 104 |  |  |  |  |  |  |  |  |  |
| 105 |  |  |  |  |  |  |  |  |  |
| 106 |  |  |  |  |  |  |  |  |  |
| 107 |  |  |  |  |  |  |  |  |  |
| 108 |  |  |  |  |  |  |  |  |  |

| 1. **General Information about the Infant** | | | | | |
| --- | --- | --- | --- | --- | --- |
| **Questions** | | **Choices** | | **Answer** | |
| 1. What is the phone number of the persons in charge of the infant’s diet and nutrition? | | First-in-charge：**________________**  Second-in-charge：**________________**  others：**________________** | | | |
| 1. How old is the baby in month? | | months | |  | |
| 1. What’s the infant’s gender? | | 1=male，2=female | |  | |
| 1. What’s the nationality of the infant? | | 1=Han，2=Mongol，3=Hui，4=others，please illustrate | |  | |
| 1. This infant is the _________ （first, second…etc）child of the mother？ | |  | |  | |
| 1. What is the gestational age of the infant in weeks? (show birth certificate) | | **_____**weeks**____**days | | | |
| 1. Is the infant a premature one? | | 1=yes，2=no，3=I don’t know | |  | |
| 1. How is the baby delivered? | | 1=natural delivery，2=caesarean，3=I am not clear | |  | |
| 1. Where was the baby born? | | 1=city/county hospital，2=town hospital，3=family planning service agency，4=home，  5=others，lease illustrate，6=unclear | |  | |
| 1. The height of the baby at birth(show birth certificate) | | centimeters | |  | |
| 1. The weight of the baby at birth(show birth certificate) | | kilos | |  | |
| 1. **Feeding information of the infant** | | | | | |
| **Questions** | | **choices** | | **answer** | |
| 1. Is the baby fed with breast milk? | | 1=yes，2=no | |  | |
| 1. How long has the breast milk feeding last? | | months | |  | |
| 1. Is the baby fed solely with breast milk now? | | 1=yes，2=no | |  | |
| 1. How long has the baby been fed solely with breast milk? | | months | |  | |
| 1. How long has the baby been fed with milk powder (not baby-formula)? | | months | |  | |
| 1. How many milk powder did the baby eat yesterday in Mls? (not baby-formula) | | Mls | |  | |
| 1. How long has the baby been fed with baby-formula milk powder? | | months | |  | |
| 1. How many baby-formula milk powder did the baby eat yesterday in Mls? | | Mls | |  | |
| 1. At what age in month was the baby fed with supplementary food? | | months | |  | |
| 1. How many times did the baby eat porridge, steamed bread, rice,etc.? | | times | |  | |
| 1. How many times did the baby eat baby-formula (nutritious) rice flour? | | times | |  | |
| 1. Last week how many times the infant eat eggs? | | times | |  | |
| 1. Last week how many times the infant eat meat (or smashed meat/meat soup)? | | times | |  | |
| 1. Last week how many times the infant eat vegetables (or smashed vegetables)? | | times | |  | |
| 1. Last week how many times the infant eat fruit (or smashed fruit) ？ | | times | |  | |
| 1. Have you supplement calcium for the infant? | | 1=yes，2=no，3=I don’t know | |  | |
| 1. How many times did you supplement calcium for your baby **last week**? | | times | |  | |
| 1. Have you ever supplemented vitamin A and D for the infant? | | 1=yes，2=no，3=I don’t know | |  | |
| 1. How many times did you supplement vitamin A and D for your baby **last week?** | | times | |  | |
| 1. Have you ever supplement iron for the infant? | | 1=yes，2=no，3=I don’t know | |  | |
| 1. How many times did you supplement iron for your baby **last week?** | | times | |  | |
| 1. Have you ever supplement zinc for the infant? | | 1=yes，2=no，3=I don’t know | |  | |
| 1. How many times did you supplement zinc for your baby **last week?** | | times | |  | |
| 1. Have you ever supplemented other vitamins and microelements? | | 1=yes，2=no，3=I don’t know | |  | |
| 1. How many times did you supplement other vitamins and microelements for your baby **last week?** | | times | |  | |
| 1. In the **recent month**, how much did you spend in total on the purchase of vitamins and microelement? | | yuan | |  | |
| 1. In the **recent month**, how much did you spend in total on the purchase of baby-formula milk powder? | | yuan | |  | |
| 1. In the **recent month**, how much did you spend in total on the purchase of other nutrition products? | | yuan | |  | |
| 1. How many times did the infant defecate **yesterday**? | | yuan | |  | |
| 1. **knowledge of infant feeding** | | | | | |
| **Questions** | | **choices** | | **Person in charge** | |
|  |  |  |  | **1st** | **2nd** |
| 1. Where did you get knowledge of infant feeding? (multiple choices) | | 1=family members，2=friends，3=village doctors，4=family planning officials，5=women’s village directors 6=other health experts，7=books，8=TV，  9=internet，10=others，please illustrate | |  |  |
| 1. Which food on the right do you think is the most important for an infant that is 6-12 months old? | | 1=baby-formula milk powder，2=breast milk，3=pure milk，4=porridge，5=none of the above | |  |  |
| 1. At what age do you think you have to feed the baby with supplementary food? | | 1=4 months later；  2=6 months later；  3=8 months later；  4=1 year later | |  |  |
| 1. Which combination of food is more nutrition balanced for baby? | | 1=solely breast milk or baby-formula milk powder；  2=breast milk/baby-formula milk powder + porridge + noodles；  3= breast milk/baby-formula milk powder + porridge + meat；  4= breast milk/baby-formula milk powder + porridge + potatoes | |  |  |
| 1. Do you think that the deficiency of microelements (such as iron, zinc, etc.) and vitamins would have influence on the growth of the baby? | | 1=has great influence；2=has some influence；  3=has no influence；4=I don’t know | |  |  |
| 1. Do you think that the deficiency of microelements (such as iron, zinc, etc.) and vitamins would have influence on the immunity of the baby? | | 1=has great influence；2=has some influence；  3=has no influence；4=I don’t know | |  |  |
| 1. Do you think that the deficiency of microelements (such as iron, zinc, etc.) and vitamins would have influence on the intelligence development of the baby? | | 1=has great influence；2=has some influence；  3=has no influence；4=I don’t know | |  |  |
| 1. Do you think that the deficiency of microelements (such as iron, zinc, etc.) and vitamins would have influence on the future studies of the baby? | | 1=has great influence；2=has some influence；  3=has no influence；4=I don’t know | |  |  |
| 1. What are the symptoms if the baby is deficient in microelements (such as iron, zinc, etc.) and vitamins | | 1=the eyeball becomes bigger；2=the head becomes bigger；  3=lags in response；4=curly hair | |  |  |
| 1. What’s your opinion on supplementing microelements and vitamins for baby who is 6-12 months old? | | 1=the baby needs it in whatever condition；  2=the baby needs what it lacks；  3=there is no need；  4=I don’t know | |  |  |
| 1. Are you willing to supplement microelements and vitamins for your baby? | | 1=yes；2=no；3=I don’t know | |  |  |
| 1. **Infant and maternal health conditions** | | | | | |
| **Questions** | | **Choices** | | **Answer** | |
| 1. Has the infant had free physical examination? | | 1=yes，2=no，3=I don’t know | |  | |
| 1. Has the infant had microelement test? | | 1=yes，2=no，3=I don’t know | |  | |
| 1. What does the infant lack as the microelement test result showed? | | 0=hasn’t taken the test，1=Vitamin A，  2=Calcium，3=Iron，4=Zinc，  5=others，please illustrate | |  | |
| 1. Was the infant discovered to have any serious diseases were discovered in past physical examination? | | 0=hasn’t done any physical examination，1=yes，  2=no，3=I don’t know | |  | |
| 1. If any serious disease was discovered, what it is?（please illustrate，please fill No if none was discovered） | |  | | | |
| 1. Have you taken any pre-delivery physical examination when you are pregnant? | | 1=yes，2=no，3=I don’t know | |  | |
| 1. I you had the physical examination, had anyone told you knowledge about nutrition? | | 0=didn’t take the physical examination，  1=yes，2=no，3=I don’t know | |  | |
| 1. Have you taken vitamins or microelements when you are pregnant? | | 1=yes，2=no，3=I don’t know | |  | |
| 1. IF you have taken any, what are those on the right column? (multiple choices) | | 1=folic acid，2=Zinc，3=Calcium，4=Iron，5=I don’t know | |  | |
| 1. **The health condition of the infant** | | | | | |
| **Questions** | | **Choices** | | **answer** | |
| 1. Did the infant have a fever in the recent month? | | 1=yes，2=no | |  | |
| 1. Did the infant cough in the recent month? | | 1=yes，2=no | |  | |
| 1. Did the infant have diarrhea in the recent month? | | 1=yes，2=no | |  | |
| 1. Did the infant feel uncomfortable because of mal-digestion in the recent month? | | 1=yes，2=no | |  | |
| 1. Did the infant have upper respiratory tract infection (like a cold) in the recent month? | | 1=yes，2=no | |  | |
| 1. How many times did the infant fall sick in the recent month? | | times | |  | |
| 1. How much did the medical treatment for the infant cost in the recent month? | | yuan | |  | |
| 1. How much of the medical expenditure on the infant was covered by the insurance? | | yuan | |  | |
| 1. **Other feeding behavior and plans of the infant’s parents (main caregivers)** | | | | | |
| **Questions** | | **choices** | | **answer** | |
| 1. What is your opinion about the county/town family planning officials? | | 1=like them very much，2=like，3=neutral，4=dislike，5=feel them dreadful，  6=never interact with them | |  | |
| 1. What is your opinion about the village family planning officials? | | 1=like them very much，2=like，3=neutral，4=dislike，5=feel them dreadful | |  | |
| 1. When would your baby usually have breakfast? | | time | |  | |
| 1. Would you visited other groups (natural villages) when you are babysitting the infant at home? | | 1=yes，2=no | |  | |
| 1. How often would you come and visit other groups (natural villages)? | | days | |  | |
| 1. How many babies aged 0-5 months would your baby play with? | | number | |  | |
| 1. Among those babies (aged 0-5 months) who play with your baby, how many of them are in your village? | | number | |  | |
| 1. How many babies aged 6-12 months would your baby play with? | | number | |  | |
| 1. Among those babies (aged 6-12 months) who play with your baby, how many of them are in your village? | |  | |  | |
| 1. How many babies aged 1-2 years would your baby play with? | |  | |  | |
| 1. Among those babies (aged 1-2 years) who play with your baby, how many of them are in your village? | |  | |  | |
| 1. The surveyor writes down the baby’s mother’s name mentioned who are in the same village as the respondent, and compare the family code with other surveyors) | |  | |  | |
| 1. How long did you spend with parents of other babies yesterday? | | hours | |  | |
| 1. How long did the infant’s mother babysitting the baby at home after it was born? | | months | |  | |
| 1. The infant’s mother prepares to babysit her baby till what age? | | months | |  | |
| 1. Is the infant’s mother at home babysitting? | | 1=yes，2=no（if No, jump to question 86） | |  | |
| 1. If there is someone at home babysitting for you (the baby’s mother), how high need the payment be to make you choose working outside over babysitting at home?**（after this question, jump to question 87）** | | 1=2000 or below，2=2001-3000 yuan，3=3001-4000 yuan,4=4001-5000 yuan，5=5000 yuan and above，6=won’t go outside to work no matter how high is the salary | |  | |
| 1. What is the mother’s salary？ | | 1=2000 or below，2=2001-3000 yuan，3=3001-4000 yuan,4=4001-5000 yuan，5=5000 yuan and above | |  | |
| 1. Do the parents of the infant have plan of bringing the baby to the working place? | | 1=yes，2=no（If No, jump to question 89） | |  | |
| 1. If there is a plan, at what age of the baby the parents plan to bring the infant with them? | | Years old | |  | |
| 1. How many text messages could your cellphone receive in the recent week? | |  | |  | |
| 1. How would you treat the text messages that you received in the recent week? | | 1=read every message，2=read most of the messages，  3=read one or two，4=read none of them | |  | |
| 1. **General information about the infant’s family** | | | | | |
| **Questions** | | **choices** | | **answer** | |
| 1. What is the distance of your home from the village committee? | | meters | |  | |
| 1. Is your family eligible for rural low-income subsidy? | | 1=yes，2=no | |  | |
| 1. What is your approximate annual household income? | | yuan | |  | |
| 1. What is the build-up area of your house? | | Square meters | |  | |
| 1. What is the current value of your house (in 10,000 yuan)? | | 1=within 1，2=1- 5，3=5-10，4=10-30，5=30 and above | |  | |
| 1. Is your house equipped with tap water? | | 1=yes，2=no | |  | |
| 1. Is your house equipped with flush toilet? | | 1=yes，2=no | |  | |
| 1. Is your house equipped with water heater? | | 1=yes，2=no | |  | |
| 1. Does your family have washing machine? | | 1=yes，2=no | |  | |
| 1. Does your family have computer? | | 1=yes，2=no | |  | |
| 1. Does your family have internet connection? | | 1=yes，2=no | |  | |
| 1. Does your family have refrigerator? | | 1=yes，2=no | |  | |
| 1. Does your family have air conditioner? | | 1=yes，2=no | |  | |
| 1. Does your family have motor vehicle/electrocar? | | 1=yes，2=no | |  | |
| 1. Does your family have car/truck? | | 1=yes，2=no | |  | |
|  | | | | | |
| 1. **Questionnaire for age and growth process (applicable to infants of 3-8 months old)** | | | | | |
| **I would ask you questions about the baby’s behaviors. For each behavior, please choose the option that best fits your baby. ( most of the time, sometimes, seldom or none) or whether such behavior is worrying you.** | | | | | |
| **Question** | **options** | **Answer** | **question** | **options** | **answer** |
| 1. When you baby was disturbed, would he be quiet within half an hour? | 1=most of the time yes  2=sometimes yes  3=seldom or never it is the case |  | Is such behavior worrying you? | 1=yes  2=no |  |
| 1. Would your baby smile at you and other family members? | 1=most of the time yes  2=sometimes yes  3=seldom or never it is the case |  | Is such behavior worrying you? | 1=yes  2=no |  |
| 1. Does your baby like being held? | 1=most of the time yes  2=sometimes yes  3=seldom or never it is the case |  | Is such behavior worrying you? | 1=yes  2=no |  |
| 1. Would your baby become rigid and lean backward when you hold it? | 1=most of the time yes  2=sometimes yes  3=seldom or never it is the case |  | Is such behavior worrying you? | 1=yes  2=no |  |
| 1. When you are talking with your baby, would he watch you and appear that he is listening? | 1=most of the time yes  2=sometimes yes  3=seldom or never it is the case |  | Is such behavior worrying you? | 1=yes  2=no |  |
| 1. Are there some behaviors of your baby that tell you it is sick or hungry? | 1=most of the time yes  2=sometimes yes  3=seldom or never it is the case |  | Is such behavior worrying you? | 1=yes  2=no |  |
| 1. When the baby is awake, does he look like enjoying seeing the adult or listening to what the adult was saying? | 1=most of the time yes  2=sometimes yes  3=seldom or never it is the case |  | Is such behavior worrying you? | 1=yes  2=no |  |
| 1. Can your baby become quiet by itself? (such as by sucking its fingers) | 1=most of the time yes  2=sometimes yes  3=seldom or never it is the case |  | Is such behavior worrying you? | 1=yes  2=no |  |
| 1. Would your baby cry for a long time? | 1=most of the time yes  2=sometimes yes  3=seldom or never it is the case |  | Is such behavior worrying you? | 1=yes  2=no |  |
| 1. Is the baby’s body relaxed? | 1=most of the time yes  2=sometimes yes  3=seldom or never it is the case |  | Is such behavior worrying you? | 1=yes  2=no |  |
| 1. Is there any difficult for your baby to suck breast milk or feeding bottle? | 1=most of the time yes  2=sometimes yes  3=seldom or never it is the case |  | Is such behavior worrying you? | 1=yes  2=no |  |
| 1. Is the time feeding the baby exceeding half an hour? | 1=most of the time yes  2=sometimes yes  3=seldom or never it is the case |  | Is such behavior worrying you? | 1=yes  2=no |  |
| 1. Do both you and your baby like the process of feeding?(including both of breast feeding and feeding-bottle) | 1=most of the time yes  2=sometimes yes  3=seldom or never it is the case |  | Is such behavior worrying you? | 1=yes  2=no |  |
| 1. Is there any difficult for your baby to eat? (such as choking and vomiting) | 1=most of the time yes  2=sometimes yes  3=seldom or never it is the case |  | Is such behavior worrying you? | 1=yes  2=no |  |
| 1. During the daytime can your baby keep awake for above an hour? | 1=most of the time yes  2=sometimes yes  3=seldom or never it is the case |  | Is such behavior worrying you? | 1=yes  2=no |  |
| 1. Is there any difficult for your baby to fall asleep at noon or at night? | 1=most of the time yes  2=sometimes yes  3=seldom or never it is the case |  | Is such behavior worrying you? | 1=yes  2=no |  |
| 1. Can your baby have at least 10 hours of sleep within 24 hours? | 1=most of the time yes  2=sometimes yes  3=seldom or never it is the case |  | Is such behavior worrying you? | 1=yes  2=no |  |
| 1. Is your baby having constipation or diarrhea? | 1=most of the time yes  2=sometimes yes  3=seldom or never it is the case |  | Is such behavior worrying you? | 1=yes  2=no |  |
| 1. Have anyone else showed concern for your baby’s behaviors? | 1=most of the time yes  2=sometimes yes  3=seldom or never it is the case |  | Is such behavior worrying you? | 1=yes  2=no |  |
| 1. If you chose “sometimes yes” or “most of the time yes”, please explain the reasons here. |  | | | | |
| 1. Are you worried about your baby’s dietary and sleeping behavior? If so, please explain. |  | | | | |
| 1. Is there anything that you worried about your baby? If yes, please explain. |  | | | | |
| 1. What are you most happy about your baby? |  | | | | |

| 1. **questionnaire for age and growth process (applicable for 9-14 months old babies)** | | | | | |
| --- | --- | --- | --- | --- | --- |
| **I would ask you questions about the baby’s behaviors. For each behavior, please choose the option that best fits your baby. (most of the time, sometimes, seldom or none) or whether such behavior is worrying you.** | | | | | |
| **Questions** | **Options** | **answer** | **Questions** | **options** | **answer** |
| 1. Would your baby smile or laugh towards you and your family members? | 1=most of the time yes  2=sometimes yes  3=seldom or never it is the case |  | Is such behavior worrying you? | 1=yes  2=no |  |
| 1. Would your baby come to find you if it is approached by strangers? | 1=most of the time yes  2=sometimes yes  3=seldom or never it is the case |  | Is such behavior worrying you? | 1=yes  2=no |  |
| 1. Does your baby like playing at places where there are families and friends? Or does your baby enjoy stay with them? | 1=most of the time yes  2=sometimes yes  3=seldom or never it is the case |  | Is such behavior worrying you? | 1=yes  2=no |  |
| 1. Does your baby enjoy being held? | 1=most of the time yes  2=sometimes yes  3=seldom or never it is the case |  | Is such behavior worrying you? | 1=yes  2=no |  |
| 1. When you baby was disturbed, would he be quiet within half an hour? | 1=most of the time yes  2=sometimes yes  3=seldom or never it is the case |  | Is such behavior worrying you? | 1=yes  2=no |  |
| 1. Would your baby become rigid and lean backward when you hold it? | 1=most of the time yes  2=sometimes yes  3=seldom or never it is the case |  | Is such behavior worrying you? | 1=yes  2=no |  |
| 1. Does your baby like playing games such as hide-and-seek? | 1=most of the time yes  2=sometimes yes  3=seldom or never it is the case |  | Is such behavior worrying you? | 1=yes  2=no |  |
| 1. Is the baby’s body relaxed? | 1=most of the time yes  2=sometimes yes  3=seldom or never it is the case |  | Is such behavior worrying you? | 1=yes  2=no |  |
| 1. Would your baby cry, scream, or be angry for a long time? | 1=most of the time yes  2=sometimes yes  3=seldom or never it is the case |  | Is such behavior worrying you? | 1=yes  2=no |  |
| 1. Can your baby become quiet by itself? (such as by sucking its fingers) | 1=most of the time yes  2=sometimes yes  3=seldom or never it is the case |  | Is such behavior worrying you? | 1=yes  2=no |  |
| 1. Is you baby interested in the surroundings? Such as other people, toys, and food? | 1=most of the time yes  2=sometimes yes  3=seldom or never it is the case |  | Is such behavior worrying you? | 1=yes  2=no |  |
| 1. Does the time that you feed the baby exceed half an hour? | 1=most of the time yes  2=sometimes yes  3=seldom or never it is the case |  | Is such behavior worrying you? | 1=yes  2=no |  |
| 1. Do both you and your baby like the process of feeding?(including both of breast feeding and feeding-bottle) | 1=most of the time yes  2=sometimes yes  3=seldom or never it is the case |  | Is such behavior worrying you? | 1=yes  2=no |  |
| 1. Is there any difficult for your baby to eat? (such as choking and vomiting) | 1=most of the time yes  2=sometimes yes  3=seldom or never it is the case |  | Is such behavior worrying you? | 1=yes  2=no |  |
| 1. Is there any difficult for your baby to fall asleep at noon or at night? | 1=most of the time yes  2=sometimes yes  3=seldom or never it is the case |  | Is such behavior worrying you? | 1=yes  2=no |  |
| 1. Would your baby babble or learnt how to speak? (If it makes such sounds frequently, then choose 1) | 1=most of the time yes  2=sometimes yes  3=seldom or never it is the case |  | Is such behavior worrying you? | 1=yes  2=no |  |
| 1. Can your baby have at least 10 hours of sleep within 24 hours? | 1=most of the time yes  2=sometimes yes  3=seldom or never it is the case |  | Is such behavior worrying you? | 1=yes  2=no |  |
| 1. Is your baby having constipation or diarrhea? | 1=most of the time yes  2=sometimes yes  3=seldom or never it is the case |  | Is such behavior worrying you? | 1=yes  2=no |  |
| 1. Are there some of your baby’s behaviors that make you think that he is hungry, felt hurt, or tired? | 1=most of the time yes  2=sometimes yes  3=seldom or never it is the case |  | Is such behavior worrying you? | 1=yes  2=no |  |
| 1. When you are talking with your baby, would it turn to you, look at you or smile? | 1=most of the time yes  2=sometimes yes  3=seldom or never it is the case |  | Is such behavior worrying you? | 1=yes  2=no |  |
| 1. Would your baby attempt to hurt other babies, adults, or animals (such as through kicking or biting)? | 1=most of the time yes  2=sometimes yes  3=seldom or never it is the case |  | Is such behavior worrying you? | 1=yes  2=no |  |
| 1. Has anyone else showed concern about your baby’s behaviors? | 1=most of the time yes  2=sometimes yes  3=seldom or never it is the case |  | Is such behavior worrying you? | 1=yes  2=no |  |
| 1. If you chose “sometimes yes” or “most of the time yes”, please explain the reasons here. |  | | | | |
| 1. Are you worried about your baby’s dietary or sleeping behaviors? If yes, please explain. |  | | | | |
| 1. Are there anything about your baby that is worrying you? If yes, please explain. |  | | | | |
| 1. What are you most happy about your baby? |  | | | | |
