# Supplementary material for: Using daily text messages to improve adherence to infant micronutrient powder (MNP) packets in rural western China: A cluster-randomized controlled trial
Source: PLoS One. 2018 Jan 19;13(1):e0191549. doi: 10.1371/journal.pone.0191549 (PMC5774801; doi:10.1371/journal.pone.0191549)
Supplement: S2 File — (DOCX) [file pone.0191549.s004.docx]

**Baby Nutrition Program: Endline Questionnaire for parents**

_____city（district）_____county_______town_____village_____group（natural village），infant’s name_________；name of infant’s father：_________；name of infant’s mother：__________；survey date:_2013 year __ month ___ day，

Group:_____________name of the surveyor:_____________，questionnaire code：**□□□□□□□**

| 1. **General information of the first round tracking after the baseline survey in 2013.04** | | | |
| --- | --- | --- | --- |
| **Questions** | | **Options** | **Answer** |
| 1. The relation of the person first-in-charge of the baby’s diet and nutrition. | | 1=father，2=mother，3=grandpa on father’s side，  4=grandma on mother’s side，5=grandpa on mother’s side，6=grandma on mother’s side，  7=uncle（aunt），8=others(**please illustrate**) |  |
| 1. The relation of the person second-in-charge of the baby’s diet and nutrition. | | 1=father，2=mother，3=grandpa on father’s side，  4=grandma on mother’s side，5=grandpa on mother’s side，6=grandma on mother’s side，  7=uncle（aunt），8=others(**please illustrate**) |  |
| 1. What is the capacity of the person surveyed in terms of the baby’s diet and nutrition? | | 1=first-in-charge，2=second-in-charge，  3=others，please illustrate |  |
| 1. The relation of the person surveyed with the baby | | 1=father，2=mother，3=grandpa on father’s side，  4=grandma on mother’s side，5=grandpa on mother’s side，6=grandma on mother’s side，  7=uncle（aunt），8=others(**please illustrate**) |  |
| 1. Has the infant and the parents received the tracking survey? | | 1=yes（jump to question 8），2=no |  |
| 1. The reason why they didn’t received the tracking survey? | | 1=work outside with parents in the city，2=absent from home temporally(such as wen to the relatives’ home)，3=the parents are no longer willing to participate in the program，4=others，please illustrate |  |
| 1. If the infant are brought along with parents who work in the city, where did they go? | | Please fill out the location, and then end the survey |  |
| 1. **The feeding information of the nutrition package of the babies since intervention** | | | |
| **Questions** | | **options** | **answer** |
| 1. Is the baby sick or felt uncomfortable while doing the survey? | | 1=yes，2=no |  |
| 1. Do we need to distribute nutrition supplement package to this family? | | **1=yes，2=no（if No, then jump to question 16）** |  |
| 1. Was the baby distributed with nutrition package in 2013.04? | | 1=yes，2=no |  |
| 1. From 2013.04 till now, how many empty bags of nutrition package were collected? | | bags |  |
| 1. From 2013.04 till now, how many bags of nutrition packages were left unfed at home? | | bags |  |
| 1. After the distribution of nutrition package in 2013.04, how many bags of nutrition package have you fed the baby in total? | | bags |  |
| 1. How many bags of nutrition package did you distribute to the parents this time? （filled by the surveyor）？ | | bags |  |
| 1. Have you fed other babies within the family with the nutrition supplement package? | | 1=yes，2=no |  |
| 1. Since 2013.04, have you received free nutrition packages from other agencies? | | 1=yes，2=no |  |
| 1. Which agency distributed those nutrition supplement packages to you? | | 1=the women’s union，2=public health department，3=other agencies（please illustrate），4=I don’t know |  |
| 1. Which kind of nutrition supplement package did you feed your baby? | | 1=the one that we distributed，2=the one distributed by other agencies |  |
| 1. **Attitudes and opinions about the nutrition supplement package (this part is only applicable to those whom we need to distribute the nutrition packages to)** | | | |
| **Questions** | | **options** | **answer** |
| 1. What is the attitude of your baby towards the nutrition supplement package? | | 1=like to eat it，2=neutral，3=doesn’t like to eat it |  |
| 1. How do you usually feed your baby with the nutrition package? | | 1=mixed it with milk(powder)，2=add it in the water，3=add it I other beverages, 4=add it in other foods of the baby，5=pour it directly into the baby’s mouth，6=others，please illustrate it |  |
| 1. The feeding of the baby was ____ after giving the baby nutrition supplement package. | | 1=easier，2=unchanged，3=more difficult |  |
| 1. Are you worried about feeding the baby with the nutrition package when you first got it? | | 1=yes，2=no |  |
| 1. If you were worried, what were you worried about? | | 1=the quality of the nutrition package，2=the safety of the nutrition package，3=the baby was too little to have it，4=others，please illustrate |  |
| 1. Are you worried about feeding the baby with the nutrition package now? | | 1=yes，2=no |  |
| 1. If you are worried, what are you worried about? | | 1=the quality of the nutrition package，2=the safety of the nutrition package，3=the baby was too little to have it，4=others，please illustrate |  |
| 1. Are you feeding the baby with the nutrition package every day now? | | 1=yes， 2=no |  |
| 1. Are there any responses of the baby worrying you after it took the nutrition package? | | 1=yes，2=no，3=I don’t know |  |
| 1. If there are, what are those responses? | |  | |
| 1. Were there anything that make you don’t want to feed your baby with nutrition package since the last time you were distributed with it? | | 1=yes， 2=no（if No, jump to question 42） |  |
| 1. What are the reasons that you don’t want to feed the baby with nutrition package? | | 1=the baby’s parents didn’t allow，2=the baby was sick or didn’t feel well，3=the baby didn’t like it，  4=it is too troublesome to feed the baby with it every day，5=the baby was not at home or I forgot，  6=other people said that the baby shouldn’t be fed with it，7=others，**please illustrate** |  |
| 1. Are there anything that make you decide to feed your baby with nutrition package every day since the last time you were distributed with it? | | 1=yes， 2=no（If NO, jump to question 44） |  |
| 1. What are the things that make you decide to feed the baby with the nutrition package every day? | | 1=I or the family members felt that the baby became better after having the nutrition package，2=relatives or friends felt that the baby became better after having the nutrition package，3=other babies within the village became better after having the nutrition package，4=others，**please illustrate** |  |
| 1. Are there any other babies within the village that are having nutrition packages, as well? | | 1=yes， 2=no， 3=I don’t know |  |
| 1. Have you exchanged experience of taking the nutrition package last week with other parents? | | 1=yes，  2=no（if No, jump to question 47） |  |
| 1. Where do those parents that exchanged experience with you come from? | | 1=the same group，2=within the village，3=outside of the village |  |
| 1. Do you think there are any changes of the baby after it took the nutrition package? | | 1=it becomes better，2=it has no changes，3=it becomes worse |  |
| 1. Have you ever heard any news saying a baby gets better after taking the nutrition package? | | 1=yes， 2=no |  |
| 1. Have you ever heard any news saying a baby gets worse after taking the nutrition package? | | 1=yes， 2=no |  |
| 1. Are you willing to continue to feed your baby with the nutrition package? | | 1=yes， 2=no |  |
| 1. **The feeding information of the baby** | | | |
| **question** | | **Options** | **answer** |
| 12.0 When did the baby took breast milk since it was born? (breast milk refers to milk of the mother or other people)？ | | 1=within an hour，2=after an hour，3=have never had breast milk |  |
| 1. Is the baby still being fed with breast milk? | | 1=yes，2=no |  |
| 1. The breast feeding for the baby lasts for how long? | | months |  |
| 13.1 How many times the baby was fed with breast milk yesterday? | | times |  |
| 13.2 On average how long the baby was fed with breast milk yesterday? | | mins |  |
| 1. Is the bay fed with solely breast milk？ | | 1=yes，2=no |  |
| 14.1 Did the baby have cooled down boiled water, juice, or soup (porridge)? | | 1=yes，2=no |  |
| 14.2 Did the baby have yogurt yesterday? | | 1=yes，2=no |  |
| 14.3 How many time did the baby have yogurt yesterday? | | times |  |
| 14.4 Did the baby have other beverages yesterday? | | 1=yes，2=no |  |
| 1. How long has the baby been fed solely with breast milk? | | months |  |
| 1. How long has the baby had non-formula milk powder, bottled milk, bagged milk, or fresh milk? | | Months |  |
| 16.1how many times did the baby have non-formula milk powder, bottled milk, bagged milk, or fresh milk yesterday? | | times |  |
| 1. What is the amount of non-formula milk powder, bottled milk, bagged milk, or fresh milk that the baby had yesterday? | | Mls |  |
| 1. How long does it last for the baby to have formula-milk powder? | | months |  |
| 18.1 How old was the baby when it started to have formula-milk powder? | | month |  |
| 18.2 How many times did the baby have formula-milk powder yesterday? | | times |  |
| 1. What is the amount of the formula-milk powder that the baby had yesterday? | | Mls |  |
| 19.1 Is the brand of the formula-milk powder you bought for the baby foreign or domestic | | 1=domestic，2=foreign,3=never purchased（if 3, then jump） |  |
| 19.2 Is the formula-milk powder you bought contain iron? | | 1=yes，2=no（need to check the content of the product） |  |
| 19.3 Where did you buy the milk powder for the baby? | | 1=within the village，2=within the town，3=within the county，4=within the province，5=outside the province |  |
| 19.4 What is your opinion of the safety of the formula-milk powder? | | 1=very safe，2=safe，3=unsafe，4=very unsafe |  |
| 19.5 How much does it cost for a bottle of formula-milk powder that you bought for your baby recently? | | yuan |  |
| 19.6 How many grams of milk-powder does each bottle/bag contain? | | grams |  |
| 19.7 How long does it take for the baby to finish such as bottle/bag? | | days |  |
| 1. At what age was the baby fed with supplementary food? | | months |  |
| 1. Was the baby fed with staple food for several times yesterday? such as porridge, rice soup, steamed bread, or rice? | | 1=yes，2=no |  |
| 21.1 Did the baby eat squash, carrot, or sweet potatoes yesterday? Or food that is yellow orange inside? | | 1=yes，2=no |  |
| 21.2 Did the baby eat potatoes, Chinese yam, white carrot, or similar root vegetables yesterday? | | 1=yes，2=no |  |
| 21.3 Did the baby eat vegetables that are dark green and with leaves yesterday? | | 1=yes，2=no |  |
| 21.4 Did the baby eat persimmon, apricot, watermelon,Hami melon,tomatoes yesterday? Or fruit wthat is red or yellow? | | 1=yes，2=no |  |
| 21.5 Did the baby eat other fruit or vegetables yesterday? | | 1=yes，2=no |  |
| 21.6 Did the baby eat viscera food such as liver, lung, and heart yesterday? | | 1=yes，2=no |  |
| 21.7 Did the baby eat meat (chicken, duck, pork, lamb, etc.) yesterday? | | 1=yes，2=no |  |
| 21.8 Did the baby eat eggs yesterday? | | 1=yes，2=no |  |
| 21.9 Did the baby eat fish, shells, or sea food yesterday? | | 1=yes，2=no |  |
| 21.10 Did the baby eat beans, nuts, or any related food yesterday? | | 1=yes，2=no |  |
| 21.11 Did the baby eat cheese, yogurt, or other milk product yesterday? | | 1=yes，2=no |  |
| 21.12 Did the baby eat oil (including oil used to fry the vegetables), fat meant, or other fat-contained food yesterday? | | 1=yes，2=no |  |
| 21.13 Did the baby eat cookies, pastries, candies, chocolate, cake, etc. yesterday? | | 1=yes，2=no |  |
| 21.14 Did the baby have seasoning contained food yesterday? Such as chilies, ginger, onions, peppers, etc.) | | 1=yes，2=no |  |
| 21.15 Did the baby eat any solid (such as rice, stead bread), semi-solid (such as thick porridge), or soft (such as smashed fruit or vegetables) yesterday? | | 1=yes，2=no |  |
| 21.16 How many times did the baby eat the solid, semi-solid, or soft food yesterday | | times |  |
| 1. Have you supplemented calcium for the baby since 2015.05? | | 1=yes，2=no，3=I don’t know |  |
| 1. How many times did you supplement calcium for your baby last week? | | times |  |
| 1. Have you supplemented vitamin A and D for the baby since 2015.05? | | 1=yes，2=no，3=I don’t know |  |
| 1. How many times did you supplement vitamin A and D for your baby last week? | | times |  |
| 1. Have you supplemented iron for the baby since 2015.05? | | 1=yes，2=no，3=I don’t know |  |
| 1. How many times did you supplement iron for your baby last week? | | times |  |
| 1. Have you supplemented zinc for the baby since 2015.05? | | 1=yes，2=no，3=I don’t know |  |
| 1. How many times did you supplement zinc for your baby last week? | | times |  |
| 1. Have you supplemented other vitamins and microelements for the baby since 2015.05? | | 1=yes，2=no，3=I don’t know |  |
| 1. In the recent month, how much did you spend in total on purchasing the vitamins and microelements for the baby? | | Yuan |  |
| 1. In the recent month, how much did you spend in total on purchasing the formula-milk powder for the baby? | | Yuan |  |
| 1. How many times did the infant defecate yesterday? | | Yuan |  |
| **III. knowledge about infant feeding** | | | |
| **Questions** | **options** | | **answer** |
| 1. What are the sources of your information on baby feeding? (multiple choices) | 1=families，2=friends，3=village doctors，4=family planning officials，5=the women director 6=other health expert，7=books，8=TV，  9=internet，10=others，please illustrate | |  |
| 1. For babies that age 12-18 months, which food on the right do you think is the most important? | 1=baby-formula milk powder，2=breast milk，3=pure milk，4=porridge，5=none of the above | |  |
| 1. At what age in months do you think the baby has to be provided with supplementary food? | 1=4 months old；  2=6 months old  3=8 months old；  4=one year old | |  |
| 1. Which combination of the foods is more nutrition balanced for babies? | 1=solely breast milk or formula-milk powder；  2=breast milk or formula milk powder + porridge + noodles；  3=breast milk or formula-milk powder + porridge + meat；  4=breast milk or formula-milk powder + porridge + potatoes | |  |
| 1. Are there any influence on the growth of the baby if it lacks microelements (such as iron, zinc, etc.)and vitamins? | 1=great influence；2=some influence；  3=non influence；4=I don’t know | |  |
| 1. Are there any influence on the immunity of the baby if it lacks microelements (such as iron, zinc, etc.)and vitamins? | 1=great influence；2=some influence；  3=non influence；4=I don’t know | |  |
| 1. Are there any influence on the intelligence development of the baby if it lacks microelements (such as iron, zinc, etc.)and vitamins? | 1=great influence；2=some influence；  3=non influence；4=I don’t know | |  |
| 1. Are there any influence on the future studies of the baby if it lacks microelements (such as iron, zinc, etc.)and vitamins? | 1=great influence；2=some influence；  3=non influence；4=I don’t know | |  |
| 1. What are the symptoms of the baby if it lacks microelements (such as iron, zinc, etc.) and vitamins? | 1=the eyeballs become bigger；2=the head becomes bigger；  3=lags in responses；4=curly hair | |  |
| 1. What is your opinion of supplementing microelements and vitamins for baby that is 12-18 months old? | 1=the baby needs it in whatever condition；  2=the baby needs what it lacks缺什么补什么；  3=there is no need for it；  4=I don’t know | |  |
| 1. **The health condition of the baby** | | | |
| **Questions** | | **Options** | **answer** |
| 52.1 Does the baby have immunization card? | | 1=yes，2=no，3=I don’t know |  |
| 52.2 Did the baby vaccinated on time? | | 1=yes，2=no（check immunization card） |  |
| 1. Did the baby have a fever in the recent month? | | 1=yes，2=no |  |
| 1. Did the baby cough within the recent month? | | 1=yes，2=no |  |
| 1. Did the baby have diarrhea in the recent month? | | 1=yes，2=no |  |
| 1. Did the baby feel uncomfortable because of mal-digestion within the recent month? | | 1=yes，2=no |  |
| 1. Did the baby have upper respiratory tract infection (such as a cold) in the recent month? | | 1=yes，2=no |  |
| 1. How many times did the baby fall sick in the recent month? | | times |  |
| 1. How much in total did you spend on medical treatment for the baby in the recent month? | | yuan |  |
| 67.1 What is the most serious disease that the baby had in the most recent month? | | illustration |  |
| 67.2 How long did that disease last (referring to the disease of the previous question)? | | days |  |
| 67.3 How much did you spend on the treatment? (referring to the most serious disease above) | | yuan |  |
| 1. **Other feeding behaviors and plans of the baby’s parents** | | | |
| **Questions** | | **Options** | **Answers** |
| 1. Would you take your baby to visit other groups (natural villages) when you are taking care of the baby at home? | | 1=yes，2=no |  |
| 1. How frequently would you visit other groups (natural villages)? | | days |  |
| 1. Would your baby usually play with several babies who are 6-12 months old? | |  |  |
| 1. How many of those babies in the above question are within the same group (natural village) as you? | |  |  |
| 1. How many babies that are 1-2 years old did your baby usually play with? | |  |  |
| 1. How many of those babies in the above question are within the same group (natural village) as you? | |  |  |
| 1. The surveyor would write down the names of the mothers of those babies mentioned within the same administrative village, and match with other surveyors afterwards. Write down the codes of those mothers’ families on the right. | |  |  |
| 1. How long did you spend with other babies’ parents yesterday? | | hours |  |
| 81.1 Did you play games with your baby using toys yesterday? | | 1=yes，2=no |  |
| 81.2 Did you tell stories to your baby yesterday? | | 1=yes，2=no |  |
| 81.3 Did you use a story book to tell the stories yesterday? | | 1=yes，2=no |  |
| 81.4 Did you teach your baby how to sing a nursery rhyme yesterday? | | 1=yes，2=no |  |
| 81.5 How long did the father live with the baby after it was born? | | months |  |
| 81.6 Is the father still living with the baby now? | | 1=yes，2=no |  |
| 1. How many months did the mother spend at home taking care of the baby after it was born? | | months |  |
| 1. Till what age in months does the mother prepare to take care of the baby at home? | | months |  |
| 1. Is the mother at home now taking care of the baby? | | 1=yes，2=no（If No, jump to question 86） |  |
| 1. If there is family members available to take care of the baby, how high should her salary be for the mother to work outside instead of taking care of the baby at home? **（after asked this question, jump to question 87）** | | 1=2000 yuan or below，2=2001-3000 yuan，3=3001-4000 yuan,4=4001-5000 yuan，5=5000 yuan and above，6=won’t go outside to work no matter how high is the salary |  |
| 1. What is the mother’s salary? | | 1=2000 yuan or below，2=2001-3000 yuan，3=3001-4000 yuan,4=4001-5000 yuan，5=5000 yuan and above |  |
| 1. Do the parents of the baby have plan to take the baby to their work place in the city? | | 1=yes，2=no（If No, jump to question 89） |  |
| 1. If there is a plan, at what age do they plan to bring the baby with them? | | years old |  |

| 1. **Questionnaire about age and growth process (applicable to babies who are 9-14 months old)** | | | | | | |
| --- | --- | --- | --- | --- | --- | --- |
| **I would ask you questions about the baby’s behaviors. For each behavior, please choose the option that best fits your baby. (most of the time, sometimes, seldom or none) or whether such behavior is worrying you.** | | | | | | |
| **Questions** | **Options** | | **Answer** | **Questions** | **Options** | **Answer** |
| 1. Would your baby smile or laugh towards you and your family members? | 1=most of the time yes  2=sometimes yes  3=seldom or never it is the case | |  | Is such behavior worrying you? | 1=yes  2=no |  |
| 1. Would your baby come to find you if it is approached by strangers? | 1=most of the time yes  2=sometimes yes  3=seldom or never it is the case | |  | Is such behavior worrying you? | 1=yes  2=否no |  |
| 1. Does your baby like playing at places where there are families and friends? Or does your baby enjoy stay with them? | 1=most of the time yes  2=sometimes yes  3=seldom or never it is the case | |  | Is such behavior worrying you? | 1=yes  2=no |  |
| 1. Does your baby enjoy being held? | 1=most of the time yes  2=sometimes yes  3=seldom or never it is the case | |  | Is such behavior worrying you? | 1=yes  2=no |  |
| 1. When you baby was disturbed, would he be quiet within half an hour? | 1=most of the time yes  2=sometimes yes  3=seldom or never it is the case | |  | Is such behavior worrying you? | 1=yes  2=no |  |
| 1. Would your baby become rigid and lean backward when you hold it? | 1=most of the time yes  2=sometimes yes  3=seldom or never it is the case | |  | Is such behavior worrying you? | 1=yes  2=no |  |
| 1. Does your baby like playing games such as hide-and-seek? | 1=most of the time yes  2=sometimes yes  3=seldom or never it is the case | |  | Is such behavior worrying you? | 1=yes  2=no |  |
| 1. Is the baby’s body relaxed? | 1=most of the time yes  2=sometimes yes  3=seldom or never it is the case | |  | Is such behavior worrying you? | 1=yes  2=no |  |
| 1. Would your baby cry, scream, or be angry for a long time? | 1=most of the time yes  2=sometimes yes  3=seldom or never it is the case | |  | Is such behavior worrying you? | 1=yes  2=no |  |
| 1. Can your baby become quiet by itself? (such as by sucking its fingers) | 1=most of the time yes  2=sometimes yes  3=seldom or never it is the case | |  | Is such behavior worrying you? | 1=yes  2=no |  |
| 1. Is you baby interested in the surroundings? Such as other people, toys, and food? | 1=most of the time yes  2=sometimes yes  3=seldom or never it is the case | |  | Is such behavior worrying you? | 1=yes  2=no |  |
| 1. Does the time that you feed the baby exceed half an hour? | 1=most of the time yes  2=sometimes yes  3=seldom or never it is the case | |  | Is such behavior worrying you? | 1=yes  2=no |  |
| 1. Do both you and your baby like the process of feeding?(including both of breast feeding and feeding-bottle) | 1=most of the time yes  2=sometimes yes  3=seldom or never it is the case | |  | Is such behavior worrying you? | 1=yes  2=no |  |
| 1. Is there any difficult for your baby to eat? (such as choking and vomiting) | 1=most of the time yes  2=sometimes yes  3=seldom or never it is the case | |  | Is such behavior worrying you? | 1=yes  2=no |  |
| 1. Is there any difficult for your baby to fall asleep at noon or at night? | 1=most of the time yes  2=sometimes yes  3=seldom or never it is the case | |  | Is such behavior worrying you? | 1=yes  2=no |  |
| 1. Would your baby babble or learnt how to speak? (If it makes such sounds frequently, then choose 1) | 1=most of the time yes  2=sometimes yes  3=seldom or never it is the case | |  | Is such behavior worrying you? | 1=yes  2=no |  |
| 1. Can your baby have at least 10 hours of sleep within 24 hours? | 1=most of the time yes  2=sometimes yes  3=seldom or never it is the case | |  | Is such behavior worrying you? | 1=yes  2=no |  |
| 1. Is your baby having constipation or diarrhea? | 1=most of the time yes  2=sometimes yes  3=seldom or never it is the case | |  | Is such behavior worrying you? | 1=yes  2=no |  |
| 1. Are there some of your baby’s behaviors that make you think that he is hungry, felt hurt, or tired? | 1=most of the time yes  2=sometimes yes  3=seldom or never it is the case | |  | Is such behavior worrying you? | 1=yes  2=no |  |
| 1. When you are talking with your baby, would it turn to you, look at you or smile? | 1=most of the time yes  2=sometimes yes  3=seldom or never it is the case | |  | Is such behavior worrying you? | 1=yes  2=no |  |
| 1. Would your baby attempt to hurt other babies, adults, or animals (such as through kicking or biting)? | 1=most of the time yes  2=sometimes yes  3=seldom or never it is the case | |  | Is such behavior worrying you? | 1=yes  2=no |  |
| 1. Has anyone else showed concern about your baby’s behaviors? | 1=most of the time yes  2=sometimes yes  3=seldom or never it is the case | |  | Is such behavior worrying you? | 1=yes  2=no |  |
| 1. If you chose “sometimes yes” or “most of the time yes”, please explain the reasons here. | |  | | | | |
| 1. Are you worried about your baby’s dietary or sleeping behaviors? If yes, please explain. | |  | | | | |
| 1. Are there anything about your baby that is worrying you? If yes, please explain. | |  | | | | |
| 1. What are you most happy about your baby? | |  | | | | |

| 1. **Questionnaire of age and growth process (applicable to babies that is 15-20 months old)** | | | | | | |
| --- | --- | --- | --- | --- | --- | --- |
| **I would ask you questions about the baby’s behaviors. For each behavior, please choose the option that best fits your baby. ( most of the time, sometimes, seldom or none) or whether such behavior is worrying you.** | | | | | | |
| **Questions** | **Options** | | **Answer** | **Questions** | **Options** | **Answer** |
| 1. Would your baby look at you when you talk with him/her? | 1=most of the time yes  2=sometimes yes  3=seldom or never it is the case | |  | Is such behavior worrying you? | 1=yes  2=no |  |
| 1. Has the baby cry and scream for more than an hour when you left? | 1=most of the time yes  2=sometimes yes  3=seldom or never it is the case | |  | Is such behavior worrying you? | 1=yes  2=no |  |
| 1. Would the baby smile or laugh out when you are playing with it? | 1=most of the time yes  2=sometimes yes  3=seldom or never it is the case | |  | Is such behavior worrying you? | 1=yes  2=no |  |
| 1. Would your baby come to find you if it is approached by strangers? | 1=most of the time yes  2=sometimes yes  3=seldom or never it is the case | |  | Is such behavior worrying you? | 1=yes  2=no |  |
| 1. Is the baby’s body relaxed? | 1=most of the time yes  2=sometimes yes  3=seldom or never it is the case | |  | Is such behavior worrying you? | 1=yes  2=no |  |
| 1. Does your baby like to be held or cuddled? | 1=most of the time yes  2=sometimes yes  3=seldom or never it is the case | |  | Is such behavior worrying you? | 1=yes  2=no |  |
| 1. When the baby is unhappy, is it able to calm down within 15 minutes? | 1=most of the time yes  2=sometimes yes  3=seldom or never it is the case | |  | Is such behavior worrying you? | 1=yes  2=no |  |
| 1. Would your baby become rigid and lean backward when you hold it? | 1=most of the time yes  2=sometimes yes  3=seldom or never it is the case | |  | Is such behavior worrying you? | 1=yes  2=no |  |
| 1. Would your baby cry, scream, or being angry for a long time? | 1=most of the time yes  2=sometimes yes  3=seldom or never it is the case | |  | Is such behavior worrying you? | 1=yes  2=no |  |
| 1. Is you baby interested in the surroundings? Such as other people, toys, and food? | 1=most of the time yes  2=sometimes yes  3=seldom or never it is the case | |  | Is such behavior worrying you? | 1=yes  2=no |  |
| 1. Would your baby do a thing repeatedly and seems that it won’t stop? Such as waving hands, or rocking body, etc.? | 1=most of the time yes  2=sometimes yes  3=seldom or never it is the case | |  | Is such behavior worrying you? | 1=yes  2=no |  |
| 1. Is there any difficult for your baby to eat? (such as choking and vomiting) | 1=most of the time yes  2=sometimes yes  3=seldom or never it is the case | |  | Is such behavior worrying you? | 1=yes  2=no |  |
| 1. Is there any difficult for your baby to fall asleep at noon or at night? | 1=most of the time yes  2=sometimes yes  3=seldom or never it is the case | |  | Is such behavior worrying you? | 1=yes  2=no |  |
| 1. Do both you and your baby like the process of feeding?(including both of breast feeding and feeding-bottle) | 1=most of the time yes  2=sometimes yes  3=seldom or never it is the case | |  | Is such behavior worrying you? | 1=yes  2=no |  |
| 1. Can your baby have at least 10 hours of sleep within 24 hours? | 1=most of the time yes  2=sometimes yes  3=seldom or never it is the case | |  | Is such behavior worrying you? | 1=yes  2=no |  |
| 1. Would you baby look at the direction when you point it something? | 1=most of the time yes  2=sometimes yes  3=seldom or never it is the case | |  | Is such behavior worrying you? | 1=yes  2=no |  |
| 1. Is your baby having constipation or diarrhea? | 1=most of the time yes  2=sometimes yes  3=seldom or never it is the case | |  | Is such behavior worrying you? | 1=yes  2=no |  |
| 1. Would your baby let you know its feelings (such as being hungry, hurt, or tired) through gestures or languages? | 1=most of the time yes  2=sometimes yes  3=seldom or never it is the case | |  | Is such behavior worrying you? | 1=yes  2=no |  |
| 1. Would your baby obey simple orders? Such as sitting down? | 1=most of the time yes  2=sometimes yes  3=seldom or never it is the case | |  | Is such behavior worrying you? | 1=yes  2=no |  |
| 1. Does your baby enjoy the surrounding of families and friends, or playing with them? | 1=most of the time yes  2=sometimes yes  3=seldom or never it is the case | |  | Is such behavior worrying you? | 1=yes  2=no |  |
| 1. When you arrive at a new place (such as park or friend’s home), would the baby check that you are around? | 1=most of the time yes  2=sometimes yes  3=seldom or never it is the case | |  | Is such behavior worrying you? | 1=yes  2=no |  |
| 1. Does your baby like listening to the story or singing? | 1=most of the time yes  2=sometimes yes  3=seldom or never it is the case | |  | Is such behavior worrying you? | 1=yes  2=no |  |
| 1. Would your baby hurt itself intentionally? | 1=most of the time yes  2=sometimes yes  3=seldom or never it is the case | |  | Is such behavior worrying you? | 1=yes  2=no |  |
| 1. Does your child like playing around other kids? | 1=most of the time yes  2=sometimes yes  3=seldom or never it is the case | |  | Is such behavior worrying you? | 1=yes  2=no |  |
| 1. Would your baby attempt to hurt other babies, adults, or animals (such as through kicking or biting)? | 1=most of the time yes  2=sometimes yes  3=seldom or never it is the case | |  | Is such behavior worrying you? | 1=yes  2=no |  |
| 1. Has anyone else showed concern about your baby’s behaviors? | 1=most of the time yes  2=sometimes yes  3=seldom or never it is the case | |  | Is such behavior worrying you? | 1=yes  2=no |  |
| 1. If you chose “sometimes yes” or “most of the time yes”, please explain the reasons here. | |  | | | | |
| 1. Are you worried about your baby’s dietary or sleeping behaviors? If yes, please explain. | |  | | | | |
| 1. Are there anything about your baby that is worrying you? If yes, please explain. | |  | | | | |
| 1. What are you most happy about your baby? | |  | | | | |

| **SMS and phone changes questions (applicable only to those in the text messages intervention group parents)** | options | **Answers** |
| --- | --- | --- |
| 1. Did any family member receive text messages that reminded feeding the baby with the nutrition package? | 1=yes， 2=no（If No, jump to question 7） |  |
| 1. How many family members received the text messages that reminded feeding the baby with the nutrition package? |  |  |
| 1. How frequently did you receive the text messages? | 1=I can every day，  2=I can most of the time，  3=I can’t most of the time |  |
| 1. Would you read such messages received? | 1=yes， 2=no |  |
| 1. Would the message reminds you about feeding the baby with nutrition package? | 1=yes， 2=no |  |
| 1. Would you feel disturbed receiving the message every day from us? | 1=yes， 2=no |  |
| 1. Why didn’t you receive the messages? | 1=my phone is often turned off，2=I don’t have a phone，  3=no signal，4=I changed the phone number  5=others，please illustrate |  |
| 1. The new phone number of the person in charge of the baby’s diet and nutrition after he/she changed his/her phone number? | First-in-charge：**________________**  Second-in-charge：**________________**  others：**________________** | |
