# Supplementary material for: Using daily text messages to improve adherence to infant micronutrient powder (MNP) packets in rural western China: A cluster-randomized controlled trial
Source: PLoS One. 2018 Jan 19;13(1):e0191549. doi: 10.1371/journal.pone.0191549 (PMC5774801; doi:10.1371/journal.pone.0191549)
Supplement: S3 File — (PDF) [file pone.0191549.s005.pdf]

PROTOCOL APPLICATION FORM
Human Subjects Research
Stanford University

Title: Nourishing the Future: Improving Baby Nutrition in Rural China
Approval Period: 10/26/2012 - 10/26/2013

Protocol Director

Name		Degree (program/year if student)		Title
Scott D Rozelle		null		Senior Fellow
**Dept**	**Mail Code**	**Phone**	**Fax**	**E-mail**
FSI - FSE	6055	(650) 724-6402	null	rozelle@stanford.edu
**CITI Training current (within last 2 years for Stanford; within last year for VA)?**				Y

Admin Contact

Name		Degree (program/year if student)		Title
Alexis Medina		null		Social Science Research Asst
**Dept**	**Mail Code**	**Phone**	**Fax**	**E-mail**
FSI - FSE	6055	561-445-8685	null	amedina5@stanford.edu
**CITI Training current (within last 2 years for Stanford; within last year for VA)?**				Y

Co-Protocol Director

Name		Degree (program/year if student)		Title
**Dept**	**Mail Code**	**Phone**	**Fax**	**E-mail**
**CITI Training current (within last 2 years for Stanford; within last year for VA)?**				

Other Contact

Name		Degree (program/year if student)		Title
**Dept**	**Mail Code**	**Phone**	**Fax**	**E-mail**
**CITI Training current (within last 2 years for Stanford; within last year for VA)?**				

Faculty Sponsor

Name		Degree (program/year if student)		Title
**Dept**	**Mail Code**	**Phone**	**Fax**	**E-mail**
**CITI Training current (within last 2 years for Stanford; within last year for VA)?**				

Other Personnel

Participant Population(s) Checklist — Yes/No

- Children (under 18) — Y
- Pregnant Women and Fetuses — N

- Neonates (0 - 28 days) N
- Abortuses N
- Mentally Disabled N
- Decisionally Challenged N
- Cancer Subjects N
- Laboratory Personnel N
- Healthy Volunteers N
- Students N
- Employees N
- Prisoners N
- Other (i.e., any population that is not specified above) Y

Study Location(s) Checklist **Yes/No**

- Stanford University Y
- General Clinical Research Center (GCRC)
- Stanford Hospital and Clinics
- Lucile Packard Children's Hospital (LPCH)
- VAPAHCS (Specify PI at VA)
- Other (Click ADD to specify details) Y

Location Name	Contact Name	Contact Phone	Contact Email	Permission?	IRB?
200 villages in Northwest China	Yaojiang Shi, Professor, NW Univ. in Xian, Shaanxi	86-29-88308337	syj8882002@yahoo.com.cn	Y	N

General Checklist

Multi-site **Yes/No**

- Is this a multi-site study? A multi-site study is generally a study that involves one or more medical or research institutions in which one site takes a lead role.(e.g., multi-site clinical trial) Y
- Is Stanford the coordinating institution or are you the lead investigator for this multi-site study? Y

Site Name	Contact Name	Contact Phone	Contact Email	Permission?	IRB?

Title: Nourishing the Future: Improving Baby Nutrition in Rural China
Approval Period: 10/26/2012 - 10/26/2013

Northwest University, Xi'an, Shaanxi Province	Yaojiang Shi, Professor, School of Management	86-29-88308337	syj8882002@yahoo.com.cn	Y	N

Collaborating Institution(s) **Yes/No**

- Are there any collaborating institution(s)? A collaborating institution is generally an institution that collaborates equally on a research endeavor with one or more institutions. Y

Institution Name	Contact Name	Contact Phone	Contact Email	Permission?	IRB?
Chinese Academy of Sciences-Ctr for Chin Ag Policy	Linxiu Zhang, Deputy Director, Professor	86-13701008925	lxzhang.ccap@igsnrr.ac.cn	Y	N

Cancer Institute **Yes/No**

- Cancer-Related Studies (studies with cancer endpoints), Cancer Subjects (e.g., clinical trials, behavior/prevention) or Cancer Specimens (e.g., blood, tissue, cells, body fluids with a scientific hypothesis stated in the protocol). N

Drug /Device **Yes/No**

- Investigational drugs, biologics, reagents, or chemicals? N
- Commercially available drugs, reagents, or other chemicals administered to subjects (even if they are not being studied)? N
- Investigational Device / Commercial Device used off-label? N
- IDE Exempt Device (Commercial Device used according to label) N

- Click "yes" to confirm that you have accessed the website and read the clinicaltrials.gov reporting requirements provided.

- This study will be registered on clinicaltrials.gov?

- Protocol involves studying potentially addicting drugs? N

Tissues and Specimens **Yes/No**

- Human blood, cells, tissues, or body fluids (tissues)? Y
- Tissues to be stored for future research projects? N
- Tissues to be sent out of this institution as part of a research agreement? For guidelines, please see http://stanford.edu/group/ICO/researcher/reMTA.html http://stanford.edu/group/ICO/researcher/reMTA.html N

Biosafety (APB) **Yes/No**

- Are you submitting a Human Gene Transfer investigation using biological agent or recombinant DNA vector? If yes, please complete and attach the Gene Transfer Protocol Application Supplemental Questions to section 16 of the eProtocol application. N
- Are you submitting a Human study using biohazardous/infectious agents? If yes, refer to the http://www.stanford.edu/dept/EHS/prod/researchlab/bio/index.html Administrative Panel on BioSafety website prior to performing studies. N
- Are you submitting a Human study using samples from subjects that contain biohazardous/infectious agents? If yes, refer to the https://ehsappprd1.stanford.edu/eprobio/ Administrative Panel on BioSafety website prior to performing studies. N

Human Embryos or Stem Cells **Yes/No**

- Human Embryos or gametes? N
- Human Stem Cells (including hESC, iPSC, cancer stem cells, progenitor cells). N

Veterans Affairs (VA) **Yes/No**

- The research recruits participants at the Veterans Affairs Palo Alto Health Care System(VAPAHCS). N
- The research involves the use of VAPAHCS non-public information to identify or contact human research participants or prospective subjects or to use such data for research purposes. N
- The research is sponsored (i.e., funded) by VAPAHCS. N
- The research is conducted by or under the direction of any employee or agent of VAPAHCS (full- time, part-time, intermittent, consultant, without compensation (WOC), on-station fee-basis, on- station contract, or on-station sharing agreement basis) in connection with her/his VAPAHCS responsibilities. N
- The research is conducted using any property or facility of VAPAHCS. N

Equipment **Yes/No**

- Use of Patient related equipment? If Yes, equipment must meet the standards established by N

Hospital Instrumentation and Electrical Safety Committee (650-725-5000)

- Medical equipment used for human patients/subjects also used on animals? N
- Radioisotopes/radiation-producing machines, even if standard of care? N

Payment **Yes/No**

- Subjects will be paid for participation? See payment considerations. N

Funding **Yes/No**

- Training Grant? N
- Program Project Grant? N
- Federally Sponsored Project? N
- Industry Sponsored Clinical Trial? N

Funding

NONE

Funding - Grants/Contracts

Funding - Fellowships

Gift Funding

Dept. Funding

Other Funding

Expedited Form

A protocol must be no more than minimal risk (i.e., "not greater than those ordinarily encountered in daily life") AND must only involve human subjects in one or more of the following paragraphs.

Select one or more of the following paragraphs:

1. N **Clinical studies of drugs and medical devices only when condition (a) or (b) is met.**
   a) Research on drugs for which an investigational new drug application (21 CFR Part 312) is not required. (Note: Research on marketed drugs that significantly increases the risks or decreases the acceptability of the risks associated with the use of the product is not eligible

for expedited review.)

b) Research on medical devices for which
   i) an investigational device exemption application (21 CFR Part 812) is not required; or
   ii) the medical device is cleared/approved for marketing and the medical device is being used in accordance with its cleared/approved labeling.

2. Y **Collection of blood samples by finger stick, heel stick, ear stick, or venipuncture as follows:**

a) from healthy, nonpregnant adults who weigh at least 110 pounds. For these subjects, the amounts drawn may not exceed 550 ml in an 8 week period and collection may not occur more frequently than 2 times per week; or

b) from other adults and children, considering the age, weight, and health of the subjects, the collection procedure, the amount of blood to be collected, and the frequency with which it will be collected. For these subjects, the amount drawn may not exceed the lesser of 50 ml or 3 ml per kg in an 8 week period and collection may not occur more frequently than 2 times per week.

3. N **Prospective collection of biological specimens for research purposes by non invasive means.**

4. N **Collection of data through non invasive procedures (not involving general anesthesia or sedation) routinely employed in clinical practice, excluding procedures involving x-rays or microwaves. Where medical devices are employed, they must be cleared/approved for marketing. (Studies intended to evaluate the safety and effectiveness of the medical device are not generally eligible for expedited review, including studies of cleared medical devices for new indications.)**

Examples:

a) physical sensors that are applied either to the surface of the body or at a distance and do not involve input of significant amounts of energy into the subject or an invasion of the subject's privacy;

b) weighing or testing sensory acuity;

c) magnetic resonance imaging;

d) electrocardiography, electroencephalography, thermography, detection of naturally occurring radioactivity, electroretinography, ultrasound, diagnostic infrared imaging, doppler blood flow, and echocardiography;

e) moderate exercise, muscular strength testing, body composition assessment, and flexibility testing where appropriate given the age, weight, and health of the individual.

5. N **Research involving materials (data, documents, records, or specimens) that have been collected, or will be collected solely for nonresearch purposes (such as medical treatment or diagnosis). (NOTE: Some research in this paragraph may be exempt from the HHS regulations for the protection of human subjects. 45 CFR 46.101(b)(4). This listing refers only to research that is not exempt.)**

6. N **Collection of data from voice, video, digital, or image recordings made for research purposes.**

7. Y **Research on individual or group characteristics or behavior(including, but not limited to, research on perception, cognition, motivation, identity, language, communication, cultural beliefs or practices, and social behavior) or research employing survey, interview, oral history, focus group, program evaluation, human factors evaluation, or quality assurance methodologies. (NOTE: Some research in this category may be exempt from the HHS regulations for the protection of human subjects. 45 CFR 46.101(b)(2) and (b)(3). This listing refers only to research that is not exempt.)**

Title: Nourishing the Future: Improving Baby Nutrition in Rural China
Approval Period: 10/26/2012 - 10/26/2013

Resources:

a) Qualified staff.

Please state and justify the number and qualifications of your study staff.

Baseline and Evaluation Surveys
Principal Investigators
Scott Rozelle, PhD, Co-director of REAP, Senior Fellow, FSI, Stanford University

Linxiu Zhang, PhD, Deputy Director and Professor, Center for Chinese Agricultural Policy at Chinese Academy of Sciences; Director REAP-China.

Field directors of research:

Yaojiang Shi, PhD, Director of Northwest Socio-economic Development Research Center, Professor School of Management, Xibei University

Nutrition and Health:
Reynaldo Martorell, PhD, Robert W. Woodruff Professor of Int. Nutrition & Chair, Hubert Department of Global Health, The Rollins School of Public Health of Emory University

Specifically, Dr. Martorell is serving as a nutrition consultant to advise on specifications of the supplement powders and other elements of the design.

Other collaborators:
Zhenlin Wang, MD, Professor, Xian Jiaotong University School of Medicine will lead nursing staff in conducting all blood tests.

b) Training.

Describe the training you will provide to ensure that all persons assisting with the research are informed about the protocol and their research-related duties and functions.

Two sets of teams in the study will each get training:

Nursing team: The lead nurse is a full professor in the School of Medicine, Wang Zhenlin. Dr. Wang and Scott Rozelle will lead training of nursing teams. Dr. Wang will train on administering the hemocue fingerprick blood tests; Scott Rozelle will train team members on how to administer the IRB protocol.

Socioeconomic team: Scott Rozelle will train the socioeconomic survey team. The team will be trained in administering the survey form that will be administered to each caregiver. This form will collect information on age, gender, employment, household characteristics, and nutritional knowledge. The team will also be trained in administering the tests of child development. Scott Rozelle will train team members on how to administer the IRB protocol.

c) Facilities.

Please describe and justify.

All testing and surveying will be conducted on-site in respondents' homes, in poor rural villages located in northwest China.

d) Sufficient time.

Explain whether you will have sufficient time to conduct and complete the research. Include how much time is required.

Month 1: Preparation

Enumerators (nursing and socioeconomic teams) will be recruited and trained. Materials (including informational materials and surveys) will be completed. Trainers will be recruited and trained.

Month 2: First round of enrollment

The first round of enrollment and baseline testing occurs. All families in the sample villages with babies aged 6-9 months will be enrolled in the study and administered baseline tests.

Month 5: Second round of enrollment

The second round of enrollment and baseline testing occurs. All families in the sample villages with babies aged 6-9 months will be enrolled in the study and administered baseline tests.

Month 6: Interventions begin

Villages will be randomly assigned to one of three intervention groups or a control group:

0.) In the control group, local family planning officials will offer free information on complementary feeding to all families enrolled in the study.

1.) In the social marketing group, local family planning officials will use modern marketing techniques to educate caregivers about the micronutrient supplementation powders and offer the powders for sale for a small fee.

2.) In the free supplementation group, local family planning officials will offer free micronutrient supplementation powders to all families enrolled in the study.

3.) In the conditional cash transfer (CCT) group, local family planning officials will offer free micronutrient supplementation powders to all families enrolled in the study and will pay caregivers if they can show that they have been using the powders.

Month 11: Midline survey

All families participating in the project will be administered a midline survey and blood testing, identical to that performed at the baseline.

Month 23: Endline survey

All families participating in the project will be administered a midline survey and blood testing, identical to that performed at the baseline.

Month 24-36
Data will be entered and de-identified. Preliminary reports and analyses will be completed. Final reports and

papers will be completed.

e) **Access to target population.**

Explain and justify whether you will have access to a population that will allow recruitment of the required number of participants.

We will be working with the National Population and Family Planning Center (NPFPC), a ministerial level authoritative body, which will arrange access to the sample villages that will be chosen randomly from a list of all villages in the sample counties.

NPFPC is highly supportive of this project. They want to see improved infant and toddler health, whether achieved by better information, more readily available nutrients, or direct cash transfers.

f) **Access to resources if needed as a consequence of the research.**

State whether you have medical or psychological resources available that participants might require as a consequence of the research when applicable. Please describe these resources.

A team of trained and certified nurses from the School of Medicine, Xian Jiaotong University, under the direction of collaborator, Dr. Zhenlin Wang (Professor) will use hemocue, finger-prick testing cards to test babies' hemoglobin levels. Dr. Wang and her team will be available to address any medical problems that arise during the baseline and follow up blood test portions of the research. Dr. Wang has been involved in many previous studies like this. Most recently, she lead a team of nurses to do health checks for boarding school students for a Ford Foundation supported study led by a team of social scientists from Northwest University, Xian. She has worked for other UN groups.

Note: If in the process of conducting the blood tests our team of nurses find children who appear to have severely low hemoglobin levels (beyond the WHO standards for age and gender for severe iron deficiency), these children will immediately be removed from the study and will be referred for immediate treatment by local doctors.

g) **Lead Investigator or Coordinating Institution in Multi-site Study.**

Please explain (i) your role in coordinating the studies, (ii) procedures for routine communication with other sites, (iii) documentation of routine communications with other sites, (iv) planned management of communication of adverse outcomes, unexpected problems involving risk to participants or others, protocol modifications or interim findings.

(i) Scott Rozelle is in charge of coordinating all of the parties. Dr. Linxiu Zhang (main collaborator for research-Chinese Academy of Sciences) will be in regular contact with the enumeration and nursing teams. Rozelle and Zhang are in daily email and phone contact.
(ii) Email and cell phone
(iii) Communication diaries will be kept of all phone communications; email traffic will be stored.
(iv) If any unexpected problem arises, the enumeration and nursing teams will immediately contact Linxiu Zhang.

1. Purpose

a) **In layperson's language state the purpose of the study in 3-5 sentences.**

The goal of this research is to measure the incidence of baby undernutrition in poor, rural

areas of China, and to evaluate which of three different strategies to improve infant nutrition is most effective relative to a control group.

b) State what the Investigator(s) hope to learn from the study. Include an assessment of the importance of this new knowledge.

We propose four specific aims:

(1) To provide new estimates of anemia prevalence among babies in rural China. Very little epidemiological evidence on anemia rates among Chinese babies is available. In our previous work, we have produced preliminary estimates suggesting that prevalence rates among schoolchildren are higher than previous believed, ranging between up to 50% in many rural counties. We suspect that rates are at least as high among the younger children who will participate in this current study.

(2) To compare the behavioral responses of caregivers in rural China when faced with different sets of information and costs for nutritional supplement powders for their children, and to measure the health consequences of each type of response.

(3) To calculate the cost-effectiveness of various approaches for anemia reduction.

A better understanding of these factors is crucial given the significant potential impacts. Iron deficiencies and anemia appear to still stubbornly plague elementary school aged children in China, and we suspect that prevalence rates will be equally high among babies, if not higher. This has never been studied on such a large scale. There has been little systematic evaluative research done to test the effectiveness of different approaches to overcome the nutrition problems.

c) Explain why human subjects must be used for this project. (i.e. purpose of study is to test efficacy of investigational device in individuals with specific condition; purpose of study is to examine specific behavioral traits in humans in classroom or other environment)

The purpose of the study is determine the positive impacts on the health and development of very young children in rural China. At the same time, this study intends to uncover the human behavioral responses that enhance or undermine policy implementation to effect positive impacts on health and educational attainment of children in schools.

2. Study Procedures

a) Describe all the procedures, from screening through closeout, which the human subject must undergo in the research project, including study visits, drug treatments, randomization and the procedures that are part of standard of care.

I. Create a baseline of information
a.) Establish the nutritional status of babies in China's poor rural areas by conducting

HemoCue 201+ finger prick blood tests of all babies aged 6-9 months in our sample villages.

b.) Administer all babies in the study an age-appropriate test of infant development.

c.) Collect basic household information from caregivers.

II. Implement Interventions

a.) In the control group, local family planning officials will offer free information on complementary feeding to all families enrolled in the study.

b.) In the social marketing group, local family planning officials will use modern marketing techniques to educate caregivers about the micronutrient supplementation powders and offer
the powders for sale for a small fee. Local family planning officials will also offer the same free information on complementary feeding that is supplied in the control group.

c.) In the free supplementation group, local family planning officials will offer free micronutrient supplementation powders to all families enrolled in the study. Local family planning officials will also offer the same free information on complementary feeding that is supplied in the control group.

d.) In the conditional cash transfer (CCT) group, local family planning officials will offer free micronutrient supplementation powders to all families enrolled in the study and will pay caregivers if they can show that they have been using the powders. Local family planning officials will also offer the same free information on complementary feeding that is supplied in the control group.

Families who receive or purchase the supplementation powders will be told to mix 1 packet into their baby's porridge each day. These supplementation powders have been approved for
daily consumption (for anyone over 6 months of age) by China's national Food & Drug Administration. They are available for over the counter purchase in many areas of the country.

III. Midline survey
All baseline tests will be repeated.

IV. Endline survey
All baseline tests will be repeated.

b) Explain how the above research procedures are the least risky that can be performed consistent with sound research design.

1. The finger prick blood test is the least invasive way to determine low hemoglobin levels. Low hemoglobin levels are indicative of iron deficiency and, if severe, anemia. By using a simple finger prick blood test, we avoid doing any of the more invasive and risky procedures available to determine anemia, which include bone marrow sampling.

2. Results from testing will be recorded by testers in confidence. Later the names will be

removed from the data before the data is released to other researchers. A number will be assigned to each family. The master code with the family names will be stored in a locked filing cabinet in Beijing, in the files of Linxiu Zhang, coPI of the project.

These will not be destroyed because some day we may want to follow up and try to determine the long run impacts of the intervention (hence the need to keep a record of the student participants).

3. We have consulted with nutrition and medical experts to develop the nutrition programs that will be offered to caregivers. The supplementation powders are simple over-the-counter multivitamin supplements that are approved for daily use for babies over 6 months of age. Our nutrition experts have also approved the powders for use in this study. The powders conform almost exactly to the multi-micronutrient "sprinkles" developed by and advocated for by UNICEF.

c) **State if deception will be used. If so, provide the rationale and describe debriefing procedures. Since you will not be fully informing the participant in your consent process and form, complete an alteration of consent (in section 13). Submit a debriefing script (in section 16).**

Deception will not be used in this study.

d) **State if audio or video recording will occur. Describe what will become of the recording after use, e.g., shown at scientific meetings, erased. Describe the final disposition of the recordings.**

N/A

e) **Describe alternative procedures or courses of treatment, if any, that might be advantageous to the participant. Describe potential risks and benefits associated with these. Any standard treatment that is being withheld must be disclosed in the consent process and form. (i.e. standard-of-care drug, different interventional procedure, no procedure or treatment, palliative care, other research studies).**

The alternative is the status quo of poor nutrition. The status quo is a continuation of some higher percentage of iron deficiency anemia among babies.

f) **Will it be possible to continue the more (most) appropriate therapy for the participant(s) after the conclusion of the study?**

It will be possible for caregivers to continue providing the supplement powders to their children after the conclusion of the study, although it will not be as convenient since we will not continue to bring the supply directly into their villages. Our study does not intend to be a permanent solution to nutrition problems in rural China.

g) **Study Endpoint. What are the guidelines or end points by which you can evaluate the different treatments (i.e. study drug, device, procedure) during the study? If one proves to be clearly more effective than another (or others) during the course of a study, will the study be terminated before the projected total participant population has been enrolled? When will the study end if no important differences are detected?**

The study endpoint is after the conclusion of the endline survey, in Month 23 of the project.

3. Background

Title: Nourishing the Future: Improving Baby Nutrition in Rural China
Approval Period: 10/26/2012 - 10/26/2013

a) **Describe past experimental and/or clinical findings leading to the formulation of the study.**

Severe iron deficiency (anemia) is known to dramatically slow cognitive, behavioral, and physical development and has been identified as one of the most significant health problems internationally. Anemia is a problem that affects children of all ages, especially those in elementary school, and has severe consequences for the educational performance of students in all grades (Nokes, van den Bosch and Bundy 1998). Our own previous studies
(2008-2012) have found the rates of iron deficiency anemia among poor elementary students in NW China to be 34% on average. (Luo et al, forthcoming)

Research increasingly indicates the importance of the nutritional programming that occurs in
the first 1000 days of life. Nutritional improvements during this brief window have been shown to have large and significant effects on health and development throughout childhood
and even into adulthood.

To date, no large-scale study has been conducted to measure the prevalence of undernutrition among babies in rural China. Given our past research of school-aged children
in these areas, we suspect that it may be quite high.

We have designed the current study to measure the rates of undernutrition in this important
population, and also to identify the most effective way of addressing this undernutrition.

b) **Describe any animal experimentation and findings leading to the formulation of the study.**

N/A.

4. Radioisotopes or Radiation Machines

a) **List all standard of care procedures using ionizing radiation (radiation dose received by a subject that is considered part of their normal medical care). List all research procedures using ionizing radiation (procedures performed due to participation in this study that is not considered part of their normal medical care). List each potential procedure in the sequence that it would normally occur during the entire study. Include the total number of times each procedure may be performed.**

Procedures	Type

b) **For radioisotope projects, provide the following radiation-related information:**

Identify the radionuclide and chemical form.

For each dosage, provide the number of times the radioisotope will be administered, the route of administration, and the activity that will be administered (mCi).

Provide dosimetry information and reference the source documents (package insert, MIRD calculation, peer reviewed literature).

c) **For radiation machine projects, provide the following diagnostic procedures:**

For well-established radiographic procedures, identify the procedures and the number of times each will be performed on a single research participant.

For each radiographic procedure, provide the setup and technique sufficient to permit dose modeling. The chief technologist can usually provide this information.

For radiographic procedures that are not well-established, provide FDA status of the machine, and information sufficient to permit dose modeling.

d) For radiation machine projects, provide the following therapeutic procedures:

For a well-established therapeutic procedure, identify the area treated, dose per fraction and number of fractions. State whether the therapeutic procedure is being performed as a normal part of clinical management for the research participants's medical condition or whether it is being performed because the research participant is participating in this project.

For a therapeutic procedure that is not well-established, provide FDA status of the machine, basis for dosimetry, area treated, dose per fraction and number of fractions.

5. Devices

a) Please list in the table below all Investigational Devices (and Commercial Devices used off-label) to be used on participants

b) Please list in the table below all Commercial devices to be used on participants

6. Drugs, Reagents, or Chemicals

a) Please list in the table below all investigational drugs, reagents or chemicals to be administered to participants.

b) Please list in the table below all commercial drugs, reagents or chemicals to be administered to participants.

7. Medical Equipment for Human Subjects and Laboratory Animals

If medical equipment used for human patients/participants is also used on animals, describe such equipment and disinfection procedures.

N/A

8. Participant Population

a) **State the following: (i) the number of participants expected to be enrolled at Stanford-affiliated site(s); (ii)the total number of participants expected to enroll at all sites; (iii) the type of participants (i.e. students, patients with certain cancer, patients with certain cardiac condition) and the reasons for using such participants.**

(i) None.

(ii) In total, 1400 babies and their caregivers will be part of this study. Our sample will include 200 villages and we estimate that each village will have 7 babies in our age range (6-9 months).

(iii) Participants will be babies aged 6-9 months at the start of the study and their caregivers. These babies are at an age when nutritional improvements can have the largest impact on future health and development, and also at the age when they can first begin eating complementary foods.

b) **State the age range, gender, and ethnic background of the participant population being recruited.**

The child participants will be aged 6-9 months at the start of the study. We expect that half will be male and half will be female. All will be ethnic Chinese. We expect that the caregivers will predominantly be women, either in their 20s (mothers) or in middle-age (grandmothers).

c) **State the number and rationale for involvement of potentially vulnerable subjects in the study (including children, pregnant women, economically and educationally disadvantaged, decisionally impaired, homeless people, employees and students). Specify the measures being taken to minimize the risks and the chance of harm to the potentially vulnerable subjects and the additional safeguards that have been included in the protocol to protect their rights and welfare.**

Approximately 1400 babies will be involved in this study. These babies are at an age when nutritional improvements can have the largest impact on future health and development, and also at the age when they can first begin eating complementary foods.

In order to minimize risks, precautionary measures will include:

a) Communication between study collaborators and caregivers (to obtain consent) [as specified by Stanford University Research Compliance Office (GUI-C41 45 CFR 46.116 [OHRP] General Requirements for Informed Consent)];

b) Ensuring all nursing staff who carry out hemocue finger-prick blood testing are well-trained and enrolled in nursing programs and UNDER the direction of a certified nurse.

c) Ensuring that caregivers and village level family planning officials are informed on how to safely administer and store the multiple micronutrient powders.

d) **If women, minorities, or children are not included, a clear compelling rationale must be provided (e.g., disease does not occur in children, drug or device would interfere with normal growth and development, etc.).**

N/A

e) **State the number, if any, of participants who are laboratory personnel, employees, and/or students. They should render the same written informed consent. If payment is allowed, they should also receive it. Please see Stanford University policy at http://www.stanford.edu/dept/DoR/rph/7-5.html).**

None of the study participants will be Stanford laboratory personnel, employees, or students.

f) **State the number, if any, of participants who are healthy volunteers. Provide rationale for the inclusion of healthy volunteers in this study. Specify any risks to which participants may possibly be**

exposed. Specify the measures being taken to minimize the risks and the chance of harm to the volunteers and the additional safeguards that have been included in the protocol to protect their rights and welfare.

None.

g) **Describe how potential participants will be identified for recruitment (e.g., chart review, referral from individual's treating physician, responses to an ad). Describe how participants will be recruited and how they will initially learn about the research (e.g., clinics, advertising). If this is a clinical trial, indicate the recruitment option selected in registering the trial on the Stanford Clinical Trials web site-whether recruitment is limited to "invitation only" (e.g. your own patients), or whether recruitment will be open to the general public. Attach recruitment materials in Section #16 (Attachments). You may not contact potential participants prior to IRB approval. See guidance Advertisements: Appropriate Language for Recruitment Material.**

All families with babies aged 6-9 months at the start of the study will be invited to participate in the study. These families will be identified based on village rosters and also based on the village-level records of our collaborators at the National Population and Family Planning Commission, with whom all families are required by Chinese law to register their births.

h) **Inclusion and Exclusion Criteria.**

Identify inclusion criteria.

All babies aged 6-9 months at the start of the study (and their caregivers) will be included in the study.

Identify exclusion criteria.

We are not including rich, urban areas in the randomized selection. These areas are not known to have large nutrition problems.

Children found in the initial blood test to be severely anemic will be excluded (and sent to a doctor for treatment).

i) **Describe your screening procedures, including how qualifying laboratory values will be obtained. If you are collecting personal health information prior to enrollment (e.g., telephone screening), please request a limited waiver of authorization (in section 15).**

We are not conducting screening. We are randomly selecting villages and automatically enrolling all families with children aged 6-9 months within these villages. We are using no prior information at the individual level.

j) **Describe how you will be cognizant of other protocols in which participants might be enrolled. Please explain if participants will be enrolled in more than one study.**

Participants will not be involved in any other study.

k) **Payment. Explain the amount and schedule of payment, if any, that will be paid for participation in the study. Substantiate that proposed payments are reasonable and commensurate with the expected contributions of participants and that they do not constitute undue pressure on participants to volunteer for the research study. Include provisions for prorating payment. See payment considerations**

N/A

l) **Costs. Please explain any costs that will be charged to the participant.**

In one of our experimental arms, we will offer the nutritional packets to caregivers at a low cost. This cost will be below market price, and is put into place to test the elasticity of caregivers' demand for the nutritional supplements.

m) Estimate the probable duration of the entire study. Also estimate the total time per participant for: (i) screening of participant; (ii) active participation in study; (iii) analysis of participant data.

The entire study will last 23 months.

(i) No screening.
(ii) Active participation per participant will involve a maximum of 2 hours per survey (baseline, midline, endline), or 6 hours total.
(iii) We estimate that data cleaning and analysis will take 12 months.

9. Risks

a) For the following categories include a scientific estimate of the frequency, severity, and reversibility of potential risks. Wherever possible, include statistical incidence of complications and the mortality rate of proposed procedures. Where there has been insufficient time to accumulate significant data on risk, a statement to this effect should be included. (In describing these risks in the consent form to the participant it is helpful to use comparisons which are meaningful to persons unfamiliar with medical terminology.)

Investigational devices.

N/A.

Investigational drugs. Information about risks can often be found in the Investigator's brochure.

N/A

Commercially available drugs, reagents or chemicals. Information about risks can often be found in the package insert.

Multimicronutrient powders: No risk. This is an over the counter multivitamin supplement powder to be mixed in with the babies' porridge. It has been approved for sale and distribution by the national Chinese government.

Procedures to be performed. Include all investigational, non-investigational and non-invasive procedures (e.g., surgery, blood draws, treadmill tests).

There is only a slight risk of infection with the HemoCue 201+, finger prick blood tests. However, this test is the standard public health methodology and has been shown repeatedly to be safe. The risk is greatly mitigated as we are using trained nurses who clean the finger prior to testing. Each cuvette used is new and sterile. There is some discomfort associated with the test.

Radioisotopes/radiation-producing machines (e.g., X-rays, CT scans, fluoroscopy) and associated risks.

N/A.

Physical well-being.

Minimal risk. Hemocue tests given by trained nurses are virtually risk free.

Psychological well-being.

Minimal risk. Any psychological 'surprise' of a caregiver learning that her child is severely iron deficient (anemic) will be mitigated by an explanation of what anemia is and that it can be eliminated with treatment. The caregiver will also be referred to a doctor for treatment.

Economic well-being.

No risk.

Social well-being.

No risk. All survey and developmental test results will be kept confidential. As soon as the surveys are collected, they will immediately have all names eliminated from the survey form and the confidential list of names and codes will be kept in a locked file in Beijing (with Dr. Linxiu Zhang).

Overall evaluation of Risk.

Low - innocuous procedures such as phlebotomy, urine or stool collection, no therapeutic agent, or safe therapeutic agent such as the use of an FDA approved drug or device.

b) In case of overseas research, describe qualifications/preparations that enable you to both estimate and minimize risks to participants.

The key medical collaborator is Zhenlin Wong, MD & Professor, Xian Jiaotong University, School of Medicine, who will be overseeing the nursing teams and running the field blood tests. She will conduct the trainings (the protocol including using new and clean testing cards for each test; safe disposal of medical waste from tests; every routine medical precaution for children).

c) Describe the planned procedures for protecting against and minimizing all potential risks. Include the means for monitoring to detect hazards to the participant (and/or to a potential fetus if applicable). Include steps to minimize risks to the confidentiality of identifiable information.

All test results will be recorded in confidentiality. Any sharing of data outside of the collaborators will be done so with unique identifiers substituted for child names, to insure anonymity.

As soon as the surveys are collected, they will immediately have all names eliminated from the survey form and the confidential list of names and codes will be kept in a locked file in Beijing (with Dr. Linxiu Zhang).

d) Explain the point at which the experiment will terminate. If appropriate, include the standards for the termination of the participation of the individual participant Also discuss plans for ensuring necessary medical or professional intervention in the event of adverse effects to the participants.

The experiment will terminate at the completion of the endline survey, in month 23 of the study.

Any caregiver who becomes distressed upon learning that her child is severely iron deficient (anemic) will be able to speak with a trained nurse, who will explain what anemia is and that it can be eliminated with treatment. The caregiver will also be referred to a doctor for treatment.

e) Special Participant Populations

Children's Findings OHRP. As children are involved in your research, please select one regulatory category (46.404 through 46.407) below that your research falls under and provide the necessary rationale for this determination. See full regulation citation.

Y 46.404 Research not involving greater than minimal risk. The research must present no greater than minimal risk to children and adequate provisions must be made for soliciting the assent of the children and the permission of their parents or guardians. Please provide rationale for the above statement.

Rationale for category selected above

(a) Blood testing is routine, requiring only a finger prick. Upside to benefits is enormous – caregivers will know whether their child is anemic or not. (b) Multi-micronutrient supplementation powders with iron are a known means to improve health and address iron deficiency. While the amount of iron in a single packet is low, over time stores of iron will improve, and with them, child health. Those not iron deficient will not be hurt by the low amount of iron in the tablets and they may well benefit from the additional vitamins. Conclusion: Risks are minimized; benefits are maximized. Benefits, in large part, go to the subpopulation which bears the minimal risk.

10. Benefits

a) **Describe the potential benefit(s) to be gained by the participants or by the acquisition of important knowledge which may benefit future participants, etc.**

Iron deficiencies and anemia appear to still stubbornly plague elementary school aged children in China. According to the international literature, this can almost certainly account for part of the poor educational performance of China's rural poor. Yet, remarkably, there is little known about the true incidence and seriousness of the micronutrient deficiencies among younger children and their effect on future development. There also has been little systematic evaluative research done to test the effectiveness of different approaches to overcome the nutrition problems.

This study is expected to benefit those children receiving supplements with iron. At the same time, the scientific research results of how iron deficiency levels were reduced and development improved will be best extrapolated to the same age range being tested and we expect will be most relevant to similar at-risk children. Our study's benefits are concentrated among the children participating in our research.

11. Privacy and Confidentiality

Privacy Protections

a) **Describe how the conditions under which interactions will occur are adequate to protect the privacy interests of participants (e.g., privacy of physical setting for interviews or data collection, protections for follow-up interactions such as telephone, email and mail communications).**

Confidentiality Protections: All test results will be recorded in privacy and not shared with others.

All interactions with study participants - except for the group training sessions - will take place in participants' private households, away from strangers and other study participants.

The group training sessions will take place in a small, centrally-located office in the village. All study participants from a single village will attend the training sessions together, as a group.

Confidentiality Protections

b) **Specify the PHI (protected health information) or other individually identifiable data or specimens you will obtain, use or disclose to others.**

We will obtain individually identifiable blood test data. However, will not disclose individually identifiable data to other researchers. We will not disclose the real names in any published findings.

All testing data will be entered on a form with the child's name and survey id number. As soon as the data get back to Beijing, a copy will be put into a locked filing cabinet in the Chinese Academy of Sciences (in the office of Linxiu Zhang). The names will then be

deleted
from all electronic files.

The survey forms will be kept in a filing cabinet in Beijing, but the names will be removed.

No PHI will be brought back to Stanford.

c) **Describe how data will be maintained (e.g., paper or electronic spreadsheet, desktop computer, laptop or other portable device) and how you will maintain the confidentiality and data security, (e.g. password protected computer, encrypted files, locked cabinet and office).**

Data (with the names deleted) will be kept on i.) a laptop computer, ii.) password protected. iii.) accessible only by the research team leaders. Data will be shared with the research team members only after the names have been removed. All accessible versions of the data will have names removed.

d) **Describe how data or specimens will be labeled (e.g. name, medical record number, study number, linked coding system) or de-identified. If you are de-identifying data or specimens, who will be responsible for the de-identification? If x-rays or other digital images are used, explain how and by whom the images will be de-identified.**

The data will be coded by village. The children will be randomly assigned id numbers and their names will be removed. Linxiu Zhang in Beijing will remove the identifying names.

e) **Indicate who will have access to the data or specimens (e.g., research team, sponsors, consultants) and describe levels of access control (e.g., restricted access for certain persons or groups, access to linked data or specimens).**

The research team will have access to the deidentified dataset, without the names of individual participants.

f) **If data or specimens will be coded, describe the method in which they will be coded so that study participants' identities cannot be readily ascertained from the code.**

The data will be coded by village. The children will be randomly assigned id numbers and their names will be removed.

g) **If data or specimens will be coded, indicate who will maintain the key to the code and describe how it will be protected against unauthorized access.**

The key to the code will be kept in locked filing cabinet in the Chinese Academy of Sciences, Beijing (in the office of Linxiu Zhang). The names will be deleted from all electronic files and all hard copies of the survey forms.

h) **If you will be sharing data with others, describe how data will be transferred (e.g., courier, mail) or transmitted (e.g., file transfer software, file sharing, email). If transmitted via electronic networks, describe how you will secure the data while in transit.See http://www.stanford.edu/group/security/securecomputing/. http://www.stanford.edu/group/security/securecomputing/. Additionally, if you will be using or sharing PHI see http://hipaa.stanford.edu/policy_security.html http://hipaa.stanford.edu/policy_security.html.**

A dataset for the entire research team (and for electronic transmission) will be developed with child names removed to protect identities. Since there will not be names on any files that will be transmitted, the information will be secure in transit.

i) **How will you educate research staff to ensure they take appropriate measures to protect the privacy of participants and the confidentiality of data or specimens collected (e.g. conscious of oral and written communications, conducting insurance billing, and maintaining paper and electronic data)?**

Research team members and related staff will be trained in the testing and survey protocol to record responses/observations out of view from others; shield records from others; not to discuss the performance of individuals on tests while in the presence of others.

We will have official training sessions before the commencement of the project. The two PIs,
Dr. Zhang from the Chinese Academy of Sciences, and Dr. Rozelle from Stanford University will train BOTH the team supervisors and the socioeconomic and nursing team members.

During the survey, team leaders will maintain the aggregated data files on a password protected computer. When all data are entered they will be removed from all machines except for that of Dr. Zhang from the Chinese Academy of Sciences. These files will be password protected. A dataset for the
entire research team (and for electronic transmission) will be developed with child names removed to protect identities.

12. Potential Conflict of Interest

New PHS regulations require that financial interests must be disclosed by investigators, and those that are identified as financial conflicts of interest must be eliminated or managed prior to final approval of this protocol.

When the Personnel section of this protocol is completed, the investigators will receive an email with a link to their OPACS dashboard. In OPACS, the investigator must click on the link for this protocol and answer the Financial Interest questions.

Investigators who have not received an email form OPACS can still complete their disclosures by going to their OPACS dashboard directly at opacsprd.stanford.edu. They should contact their school's COI Manager with any issues with OPACS.

The table below displays the names of investigators, and whether they have entered their financial interest disclosure, if any, in OPACS and the status of review of conflicts of interest.

You will not be able to submit this protocol until the "Financial Interest" question has been answered in OPACS for all investigators listed in the table.

Review of this protocol by IRB will occur when all investigators listed have answered the Financial Interest question in OPACS, either Yes or No.

Approval of this protocol will only occur when all investigators who have Financial Interests have submitted their OPACS disclosure and review of the information has been completed by their COI Manager.

Note: If any changes to disclosures are made while this page is open, simply reload the page to see current information.

Investigators	Email	Has	Date Financial	Date OPACS	Date OPACS

Title: Nourishing the Future: Improving Baby Nutrition in Rural China
Approval Period: 10/26/2012 - 10/26/2013

		Financial Interest?	Interest Answered	Disclosure Submitted	Review Completed
Scott D Rozelle	rozelle@stanford.edu	N	09/18/2012	N/A	N/A

13. Consent Background

13.1 Waiver of Documentation Consent for Caregivers

Sponsor's Consent Version Number: (if any):

a) **Describe the informed consent process. Include the following.**
   i) **Who is obtaining consent? (The person obtaining consent must be knowledgeable about the study.)**
   ii) **When and where will consent be obtained?**
   iii) **How much time will be devoted to consent discussion?**
   iv) **Will these periods provide sufficient opportunity for the participant to consider whether or not to participate and sign the written consent?**
   v) **What steps are you taking to minimize the possibility of coercion and undue influence?**
   vi) **If consent relates to children and if you have a reason for only one parent signing, provide that rationale for IRB consideration.**

(i) Trained members of the field survey team will be obtaining consent. All will be trained in the details of the consent process, and will be extremely familiar with the intervention itself. (ii) Consent will be obtained orally in the caregiver's home before the start of project activities. (iii) Ten minutes will be devoted to consent discussion, including five minutes to describe the study, and five minutes to ask questions. (iv) Yes. (v) The members of the research team responsible for obtaining consent will be fully trained in the consent process. Caregivers will be notified that participation in the study is voluntary. Consent will be obtained privately. (vi) N/A

b) **What is the Procedure to assess understanding of the information contained in the consent? How will the information be provided to participants if they do not understand English or if they have a hearing impairment? See /hrpp/Chapter12.html#ch12_2 HRPP Chapter12.2 for guidance.**

The information will be conveyed to parents orally, according to a predetermined script in Chinese. (The English translation of this script is attached here.) Parents will be asked if they understand what will be required of them.

c) **What steps are you taking to determine that potential participants are competent to participate in the decision-making process? If your study may enroll adults who are unable to consent, describe (i) how you will assess the capacity to consent, (ii) what provisions will be taken if the participant regains the capacity to consent,(iii) who will be used as a legally authorized representative, and (iv) what provisions will be made for the assent of the participant.**

All caregivers, by the very nature of their role taking care of a young child, can be considered competent to participate in the decision-making process. We will ensure that they are provided with sufficient information to make an informed decision.

Select one of the following regulatory criteria for a waiver of documentation (signature) and provide a protocol-specific justification:

1) **45 CFR 46.117(c)(1). For research that is not subject to FDA regulation, the only record linking the participants and the research would be the consent document,and the principal risk would be potential harm resulting from a breach of confidentiality; each participant will be asked whether he/she wants documentation linking the participant with the research, and the participant's wishes govern.**

2) Y **45 CFR 46.117(c)(2) and 21 CFR 56.109(c)(1). Research (whether it is or is not subject to FDA**

regulation) presents no more than minimal risk of harm to participants and involves no procedures for which written consent is normally required outside of the research context.

Rationale for above selection:

The fingerprick test is virtually risk free. The nutritional supplements are safe and approved by the Chinese government for regular use. All survey instruments are benign - they include internationally approved instruments for measuring infant growth and development, and a simple questionnaire about baby care practices in the household.

14. Assent Background (less than 18 years of age)

14.1 Assent Not Applicable Assent for Babies

Please explain why assent is not applicable to this study:

The babies enrolled in this study are too young (under 2 years) to understand the research study and to give their informed assent.

15. HIPAA Background

16. Attachments

Attachment Name	Attached Date	Attached By	Submitted Date
International Research Form	09/19/2012	amedina5	
Household survey	10/15/2012	amedina5	
ROZELLE_Protocol #25734 Approval	10/26/2012	aeslinge	

Obligations

The Protocol Director agrees to:

- Adhere to principles of http://humansubjects.stanford.edu/research/documents/eval_study_designGUI03017.pdf sound scientific research designed to yield valid results.
- Conduct the study according to the protocol approved by the IRB
- Be appropriately qualified to conduct the research and be trained in Human Research protection ethical principles, regulations, policies and procedures.

Title: Nourishing the Future: Improving Baby Nutrition in Rural China
Approval Period: 10/26/2012 - 10/26/2013

- Ensure all research personnel are adequately trained and supervised
- Ensure that the rights and welfare of participants are protected including privacy and confidentiality of data
- Disclose to the appropriate departments any potential conflict of interest
- Report promptly any new information, modification, or http://humansubjects.stanford.edu/research/documents/Events-Info-Report-to-IRB_GUI03P13.pdf unanticipated problems that raise risks to participants or others
- Apply relevant professional standards.

VA Protocol Directors also certify that:

- All unanticipated internal or local SAEs, whether related or unrelated to the research, will be/have been reported to the IRB
- All subjects entered onto the master list of subjects for the study will sign/have signed an informed consent form prior to undergoing any study interactions or interventions, unless granted a waiver by the IRB.

Any change in the research protocol must be submitted to the IRB for review prior to the implementation of such change. Any complications in participants or evidence of increase in the original estimate of risk should be reported at once to the IRB before continuing with the project. Inasmuch as the Institutional Review Board (IRB) include faculty, staff, legal counsel, public members, and students, protocols should be written in language that can be understood by all Panel members. The investigators must inform the participants of any significant new knowledge obtained during the course of the research.

IRB approval of any project is for a maximum period of one year. For continuing projects and activities, it is the responsibility of the investigator(s) to resubmit the project to the IRB for review and re-approval prior to the end of the approval period. A Notice to Renew Protocol is sent to the Protocol Director 7 weeks prior to the expiration date of the protocol.

Department Chair must approve faculty and staff research that is not part of a sponsored project. VA applicants must have Division Chief or Ward Supervisor approval. E-mail the Department Chair approval to IRBCoordinator@lists.stanford.edu.

All data including signed consent form documents must be retained for a minimum of three years past the completion of the research. Additional requirements may be imposed by your funding agency, your department, or other entities. (Policy on Retention of and Access to Research Data, Research Policy Handbook,)

PLEASE NOTE: List all items (verbatim) that you want to be reflected in your approval letter (e.g., Amendment, Investigator's Brochure, consent form(s), advertisement, etc.) in the box below. Include number and date when appropriate.

Y The Protocol Director has read and agrees to abide by the above obligations.
